# Development and Evaluation of the Prognostic Nomogram to Predict Refractive Error in Patients With Primary Angle-Closure Glaucoma Who Underwent Cataract Surgery Combined With Goniosynechialysis

**DOI:** 10.3389/fmed.2021.749903

**Published:** 2021-12-15

**Authors:** Yuancun Li, Chengyao Guo, Chukai Huang, Liu Jing, Yingzi Huang, Ruiqing Zhou, Kunliang Qiu, Mingzhi Zhang

**Affiliations:** ^1^Joint Shantou International Eye Center of Shantou University, The Chinese University of Hong Kong, Shantou, China; ^2^Shantou University Medical College, Shantou, China

**Keywords:** primary angle closure, refractive error, intraocular lens, nomogram, cataract surgery

## Abstract

**Objective:** To evaluate the accuracy of different intraocular lens (IOL) power calculation formulas and develop prognostic nomograms to predict the risk of postoperative refractive error in primary angle-closure glaucoma (PACG) patients.

**Methods:** A total of 111 eyes with PACG underwent goniosynechialysis combined with phacoemulsification and IOL implantation were included. SRK/T, Barrett II, Hoffer Q, and Kane formulas were used to predict postoperative refraction. Prediction error (PE) and absolute predictive error (APE) produced by the four formulas were calculated and compared. An APE >0.50 D was defined as the event. Binary logistic regression analysis and prognostic nomogram models were conducted to investigate reliable predictors associated with postoperative refraction.

**Results:** The Kane (−0.06 D) and Barrett II (−0.07 D) formulas had mean prediction error close to zero (*p* = 0.44, *p* = 0.41, respectively). The Hoffer Q and SRK/T produced significantly myopic outcomes (*p* = 0.003, *p* = 0.013, respectively). The percentage of eyes within ± 0.5 D was 49.5% (55/111), 44.1% (49/111), 43.2% (48/111), and 49.5% (54/111), for the Kane, Barrett II, Hoffer Q, and SRK/T formula, respectively. Nomogram showed that AL had the greatest impact on the refractive outcomes, indicating a shorter preoperative AL is associated with a greater probability of refractive error event. The area under the receiver operator curve (AUC) of the nomogram for the Kane, Barrett II, Hoffer Q, and SRK/T was 0.690, 0.701, 0.708, and 0.676, respectively.

**Conclusions:** The Kane and Barrett II formulas were comparable, and they outperformed Hoffer Q and SRK/T in the total eyes with PACG receiving cataract surgery combined with goniosynechialysis. The developed nomogram models can effectively predict the occurrence of postoperative refractive error events.

## Introduction

Primary angle-closure glaucoma (PACG) is one of the leading causes of irreversible blindness, disproportionally affecting Asians (1). It has been estimated to affect more than 20 million people worldwide by 2020 (2). PACG is characterized by progressively peripheral anterior synechiae which leads to closure of the anterior chamber drainage angle with subsequently elevated intraocular pressure (IOP). It has been reported that compared with trabeculectomy, goniosynechialysis (GSL) combined with phacoemulsification and intraocular lens (IOL) implantation (phaco-IOL-GSL) could reduce peripheral anterior synechiae, remove pupillary block, and also relieve the crowded anterior chamber, which has become an effective and safe treatment option for patients with PACG with coexisting cataract (3, 4).

However, the inaccurate IOL power prediction in patients with PACG can be a significant problem resulting in unsatisfying postoperative refractive outcomes. A previous study has found that the difference between predicted and actual residual refraction was significantly larger for the PACG group than the normal control group (*p* = 0.012). Furthermore, a greater proportion of eyes with PACG presented refractive error >0.5 D compared with the normal controls, which was demonstrated in this work (5). Inappropriately chosen IOL power calculation formula (6), corneal edema which affects the accuracy of biometry measurement, ocular anatomy change, and capsular apparatus shifting after the cataract surgery maybe the reasons for the inaccurate IOL power prediction in patients with PACG (7, 8). Recently, more and more biological and clinical variables of patients with PACG have been found to be the potential risk factors associated with unsatisfying refractive outcomes (9, 10). However, few works have investigated the performance of different IOL formulas in eyes with PACG.

Therefore, this work aims to investigate the accuracy of IOL power calculation formulas in patients with PACG who underwent phaco-IOL-GSL. Secondly, we tried to assess risk factors associated with postoperative refractive error. As the nomogram is wildly used as predictive model in medicine, which can generate a particular individual probability of a clinical event by diverse variables and thereby helping clinical decision making (11), we further constructed and evaluated the prognostic nomogram models for different IOL power calculation formulas.

## Methods

This work was a retrospective study which was approved by the Ethics Committee for Human Medical Research at the Joint Shantou International Eye Center of Shantou University and the Chinese University of Hong Kong (No. 2021JSIEC07015), and all procedures were designed to conform to the tenets of the Declaration of Helsinki.

### Participants

**Figure 1:**
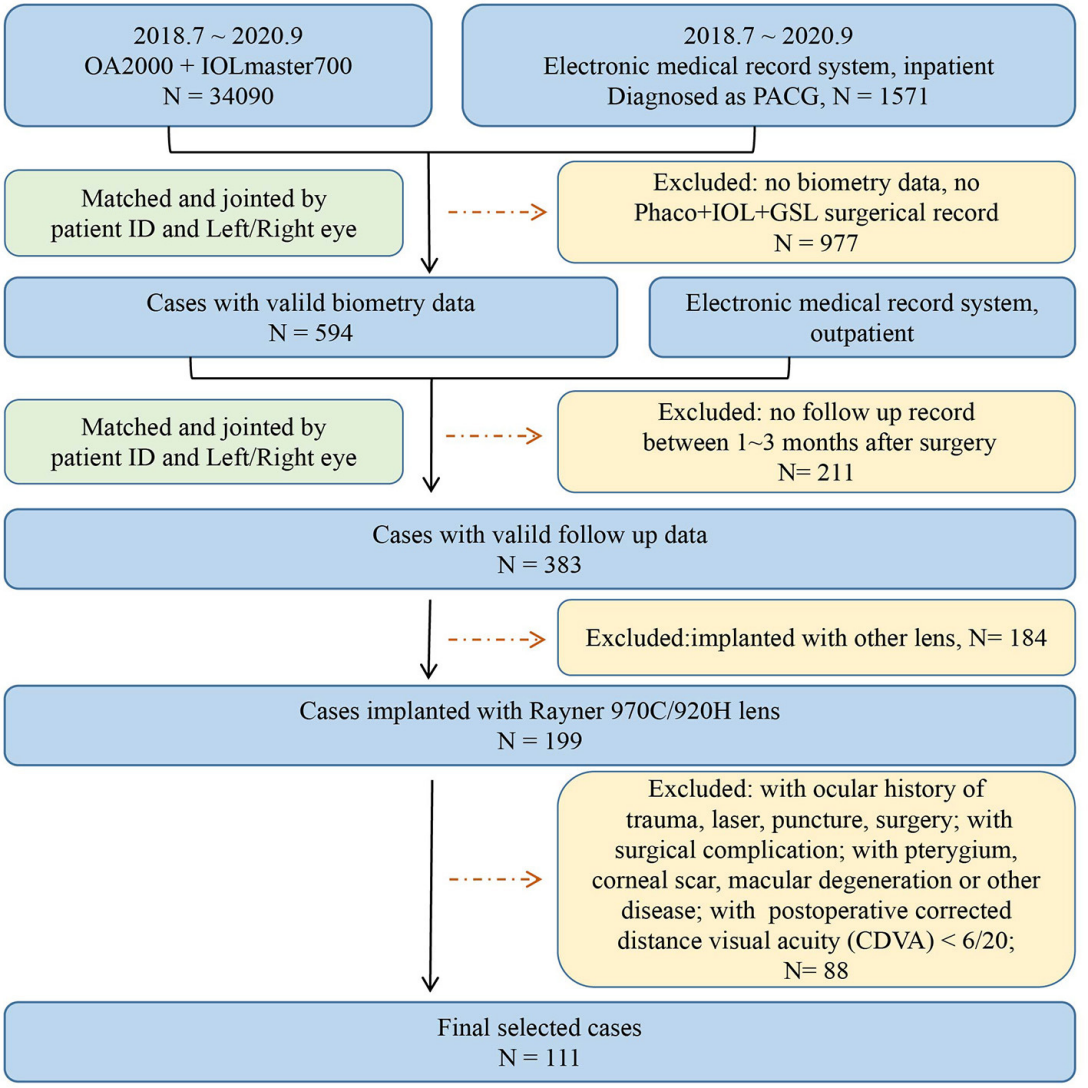
Flowchart of inclusion and exclusion of patients.

Patients diagnosed with PACG who underwent phaco-IOL-GSL from July 2018 to September 2020 were consecutively collected and reviewed. The inclusion criteria were: (1) IOL implantation using 920H and 970C IOL model form Rayner Intraocular Lenses Ltd (They are the mostly used lenses in our center, share the same material and IOL design, and were considered the same in this study); (2) cases with complete follow-up medical records; and (3) eyes with postoperative corrected distance visual acuity (CDVA) of 6/20 or more within 1–3-months. Patients with complicated cataract surgery, previous antiglaucoma surgery (such as trabeculectomy and laser peripheral iridectomy), previous corneal or vitreous surgery, acquired retinal diseases, and pathology affecting the accuracy of biometry measurement (such as pterygium, severe corneal or vitreous opacity, macular degeneration, and retinal detachment) were excluded. Only one eye of each participant was included, and the eye with better CDVA was selected if both eyes met the inclusion criteria for a particular individual. Flowchart of inclusion and exclusion of patients is available in Figure 1. All participants underwent complete ophthalmic examinations, including subjective optometry, slit-lamp biomicroscopy examination, and non-dilated indirect ophthalmoscopy examination. Ocular biometric parameters including axial length (AL), keratometry (K), anterior chamber depth (ACD), lens thickness (LT), central corneal thickness (CCT), and white-to-white (WTW) were measured by OA 2000 (Tomey Corporation, Japan) and IOL Master 700 (Carl Zeiss Meditec, Jena, Germany). All operations of selected eyes were performed by Dr. Huang, the chief of glaucoma center.

### Data Extraction

For OA2000, we used a python script which was provided by an engineer to crawl data stored in the micro-SD card of the device; for IOL master700, data can be downloaded directly from the device. Biology measurement data from different devices were pooled together for statistical analysis and modeling. Clinical data was extracted from the electronic medical record system by the help of information department of the eye center. Data was further matched and joined by patient ID and left/ right eye using R software.

### Definition of Events

SRK/T, Barrett Universal II (Barrett II), Hoffer Q, and Kane formulas were used to calculate the IOL power and predict postoperative refraction. Lens constants were from the User Group for Laser Interference Biometry (ULIB)^1^ for all formulas. The refractive prediction error (PE) was calculated by subtracting the formula-predicted postoperative refraction from the actual postoperative refraction, and the absolute predictive error (APE) was defined as the absolute value of PE. Actual postoperative refraction was defined as spherical equivalent refraction of 1–3 months after cataract surgery. Since accurate IOL power prediction was defined as APE within 0.50 diopters (D), the refractive error event was defined as APE > 0.50 D in the present work. The percentages of eyes with PE within ± 0.50, ± 0.75, and ± 1.00 D of the targeted refraction were also calculated for each formula.

### Nomogram Construction and Evaluation

The following data was collected as potential independent variables: (1) demographic characteristics including age and gender; and (2) biometric measurements including AL, K, CCT, ACD, LT, and WTW. Univariate logistic regression model was conducted to evaluate the crude relationship between refractive error events with independent variables, and then all variables underwent multivariate logistic regression analysis. Based on these analyses, a prognostic nomogram model was constructed for each formula. The performance of each model was evaluated from two perspectives: the Discrimination ability of the model was depicted by area under the receiver operator curve (AUC); accuracy of the model was depicted using Hosmer-Lemeshow goodness-of-fit test. The model was internally validated using the bootstrapping method. The source code for logistic regression analysis and nomogram construction was detailed, as described in the Supplementary Material 1.

### Statistical Analyses

Statistical analyses were performed by commercially available software (R version 4.0.2, R Foundation; Boston, MA and IBM SPSS Statistics 21; SPSS Inc., Chicago, IL). Shapiro–Wilk test was used to evaluate the normality of the continuous variable. One-sample *T*-test was used to assess whether the PE for each formula was significantly different from zero. The Friedman test was performed to assess the differences in the absolute errors among formulas, followed by the Wilcoxon signed-rank test with Bonferroni correction to assess whether there was a significant difference between formulas. The Fisher's exact test was employed to evaluate the percentage of PE within ± 0.50 D and ± 0.75 D between the formulas. R packages “regplot,” “rmda,” “rms” were used to construct the nomogram and assess the performance of the predictive model. Mean (mean ± standard deviation) values and relative risks (odds ratios with 95% confidence interval) were presented. The value *p* < 0.05 was defined as statistically significant.

## Results

A total of 111 eyes from 111 participants with a mean age of 64.21 ± 8.06 were included in is work. There were 33 (29.73%) men and 78 (70.27%) women, as well as 63 (56.76%) right eyes and 48 (40.54%) left eyes. Demographic and biometric data of the study population are summarized in Table 1.

**Table 1:** Clinical characteristics of the study population (*N* = 111).

**Parameter**	**Mean ± SD**	**Range**
Age (yr)	64.21 ± 8.06	(46, 83)
Gender, *n* (%)		
Male	33 (29.73%)	
Female	78 (70.27%)	
Eye, *n* (%)		
Right	63 (56.76%)	
Left	48 (40.54%)	
Mean K (D)	44.59 ± 1.56	(40.16, 49.10)
Flat K (D)	44.11 ± 1.56	(39.31, 48.56)
Steep K (D)	45.07 ± 1.63	(41.02, 49.63)
AL (mm)	22.42 ± 0.87	(19.68, 24.50)
ACD (mm)	2.31 ± 0.24	(1.69, 2.90)
LT (mm)	4.97 ± 0.32	(4.33, 5.78)
WTW (mm)	11.34 ± 0.48	(10.14, 12.60)
CCT (um)	552.67 ± 37.68	(475.00, 643.77)
IOL power	24.00 ± 2.38	(16.00, 30.00)
IOL model, *n* (%)		
Rayner 920H	29 (26.13%)	
Rayner 970C	82 (73.87%)	

*AL, axial length; K, keratometry; ACD, anterior chamber depth; LT, lens thickness; CCT, central corneal thickness; W2W, white to white distance; IOL, intraocular lens; D, diopter*.

### Comparison of IOL Power Calculation Formulas

**Figure 2:**
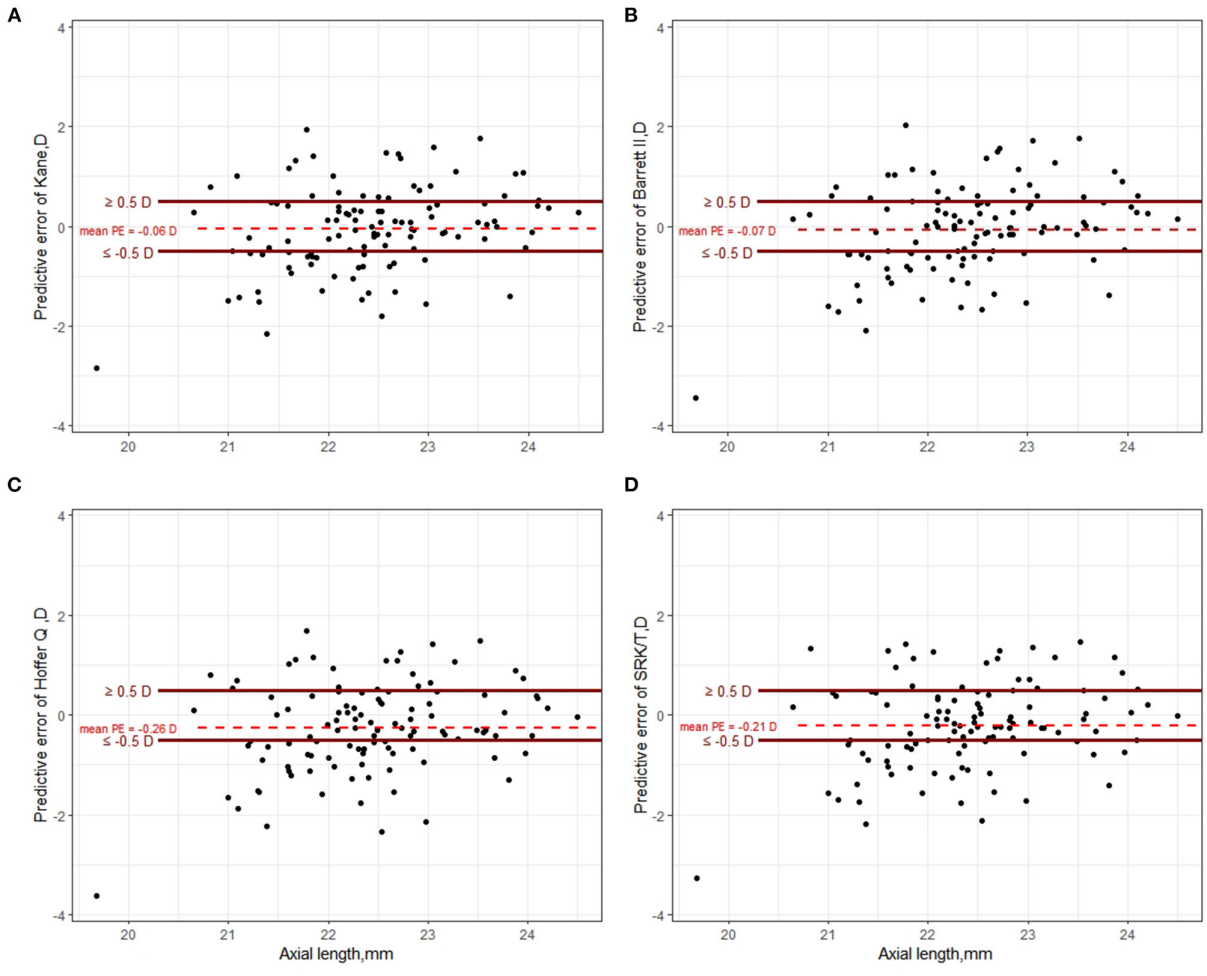
Scatter plot of prediction error of Kane **(A)**, Barrett II **(B)**, Hoffer Q **(C)**, and SRK/T **(D)** formula, respectively.

**Figure 3:**
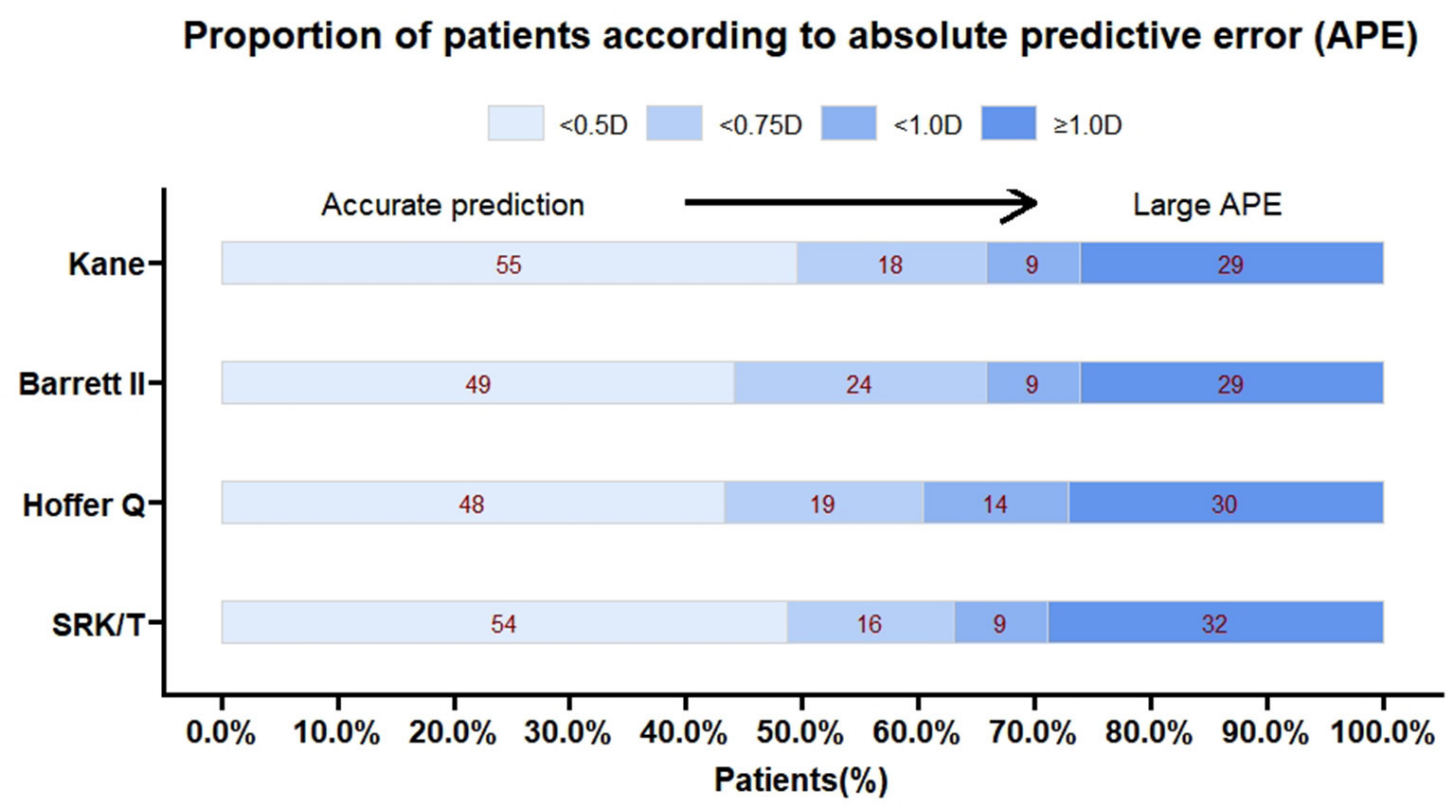
The percentage of eyes with a prediction error within ± 0.5, ± 0.75 and ± 1.00 D for each formula.

The predictive outcomes of the four formulas for all eyes are displayed in Table 2 and Figure 2. The Kane (−0.06 D) and Barrett II (−0.07 D) formulas had mean prediction error close to zero, which showed no significant difference from zero (*p* = 0.44, *p* = 0.41, respectively). The other two formulas, Hoffer Q and SRK/T produced significantly myopic outcomes (*p* = 0.003, *p* = 0.013, respectively). The MedAEs predicted by the Kane, Barrett II, Hoffer Q, and SRK/T formulas showed no significant difference (0.49 D, 0.56 D, 0.57 D, 0.51 D, respectively, *P* = 0.148). Figure 3 shows the percentages of eyes with PE within ± 0.50 D, ± 0.75 D, and ± 1.00 D of the targeted refraction with four formulas. The percentage of eyes with PE within ± 0.50 D was only slightly higher using the Kane formula (50.45%, 56/111) when compared with the other three formulas (*p* = 0.688, Fisher's exact test). As for the eyes within ± 0.75 D of the targeted refractive error, the percentage of the Kane and Barrett II formula was equal (65.77%, 73/111) and was higher than the 63.96% (71/111) of SRK/T and 60.36% (67/111) of Hoffer Q formula, but without significance (*p* = 0.831, Fisher's exact test).

**Table 2:** Refractive prediction error, mean absolute error and median absolute error produced by each formula.

**Formula**	**ME (D)^a^**	**MAE (D)**	**MedAE (D)^b^**
Kane	−0.06 ± 0.86	0.66 ± 0.55	0.49 (0.70)
Barrett II	−0.07 ± 0.89	0.58 ± 0.57	0.56 (0.80)
Hoffer Q	−0.26 ± 0.90**	0.72 ± 0.59	0.57 (0.73)
SRK/T	−0.21 ± 0.87*	0.69 ± 0.57	0.51 (0.86)
*P*-value	-	0.148	0.148

*ME, mean prediction error; MAE, mean absolute error; MedAE, median absolute error; D, diopter*.

a *One-sample T-test analysis*.

b *Friedman test analysis*.

* *P < 0.05*.

** *P < 0.01*.

### Nomogram Development for Investigating Risk Factors

There were 57 (51.4%), 62 (55.9%), 63 (56.8%), and 56 (50.5%) eyes with refractive error events for the SRK/T, Barrett II, Hoffer Q, and Kane formula, respectively. Age, gender, and the ocular biometric parameters (including AL, K, ACD, LT, CCT, and WTW) were assessed in the univariate and multivariate logistic regression analysis to identify the factors that influence PE and further construct the prognostic nomogram. Univariate binary logistic regression analysis of the risk factors for refractive error event showed that AL was significantly associated with postoperative refractive error for all the four formulas (all *p* < 0.05, Table 3). There was no significant correlation with age, sex, mean K, ACD, LT, CCT, and WTW for all the formulas. Multivariate binary logistic regression analysis revealed that a female sex was significantly associated with the refractive error event when using the Barrett II formula (odds ratio, OR = 0.37, *p* = 0.043). But for the Kane and Hoffer Q formula, only a shorter AL was significantly associated with the event, and the OR of postoperative refractive error >0.50 D in shorter AL eyes were higher than their counterparts (OR = 0.41, *P* = 0.016 for the Kane, OR = 0.37, *P* = 0.009 for the Hoffer Q, respectively). In addition, no preoperative biometric factors closely associated with the refractive outcomes were found for the SRK/T formula in this work. The ORs for all variables in each formula are presented in Table 3.

**Table 3:** Logistic regression analysis for the refractive error event risk factors.

	**Univariate**				**Multivariate (full model)**			
	**Kane**	**Barrett II**	**Hoffer Q**	**SRK/T**	**Kane**	**Barrett II**	**Hoffer Q**	**SRK/T**
Age	0.98 (0.93, 1.03)	0.99 (0.94, 103)	1.01 (0.96, 1.06)	1.00 (0.95, 1.04)	1.03 (0.98, 1.09)	0.99 (0.94, 1.05)	1.03 (0.17, 0.75)	1.00 (0.95, 1.06)
Female	1.50 (0.66, 3.47)	0.63 (0.28, 0.99)	0.95 (0.41, 2.16)	0.70 (0.30, 1.59)	0.52 (0.20, 1.32)	0.37 (0.13, 0.94)*	0.65 (0.24, 1.66)	0.52 (0.20, 1.30)
AL	1.72 (1.1, 2.81)*	0.51 (0.31, 0.81)**	0.46 (0.27, 0.75)**	0.62 (0.38, 0.97)*	0.41 (0.19, 0.82)*	0.50 (0.23, 1.01)**·**	0.37 (0.17, 0.75)**	0.51 (0.25, 1.00)**·**
Mean K	0.83 (0.64, 1.05)	1.28 (1.00, 1.67)**·**	1.25 (0.98, 1.63)**·**	1.18 (0.92, 1.51)	1.01 (0.71, 1.44)	1.04 (0.73, 1.48)	0.94 (0.65, 1.34)	1.01 (0.72, 1.42)
ACD	1.22 (0.25, 6.13)	0.71 (0.14, 3.55)	0.86 (0.17, 4.33)	0.58 (0.11, 2.86)	1.01 (0.14, 7.11)	0.75 (0.10, 5.35)	0.68 (0.09, 4.91)	0.31 (0.04, 2.08)
LT	0.62 (0.19, 2.03)	0.81 (0.24, 2.65)	0.43 (0.12, 1.42)	0.57 (0.17, 1.87)	1.49 (0.38, 6.10)	0.85 (0.21, 3.42)	0.35 (0.08, 1.41)	0.40 (0.10, 1.55)
CCT	1.00 (0.99, 1.01)	1.00 (0.99, 1.01)	1.00 (1.00, 1.01)	1.00 (0.99, 1.01)	1.00 (0.99, 1.01)	1.00 (0.99, 1.01)	1.00 (0.99, 1.01)	0.99 (0.99, 1.01)
WTW	0.94 (0.43, 2.08)	0.47 (0.20, 1.06)**·**	0.56 (0.24, 1.25)	0.87 (0.39, 1.91)	1.84 (0.69, 5.12)	0.99 (0.94, 1.05)	1.17 (0.43, 3.17)	1.58 (0.60, 4.30)

*Data was presented as odds ratio (95% confidence interval)*.

*AL, axial length; Mean K, mean keratometry; ACD, anterior chamber depth; LT, lens thickness; CCT, central corneal thickness; W2W, white to white distance*.

· *P < 0.1*,

* *P < 0.05*,

** *P < 0.01*.

*Bold values represents that they were statistically significance at a = 0.05 level*.

**Figure 4:**
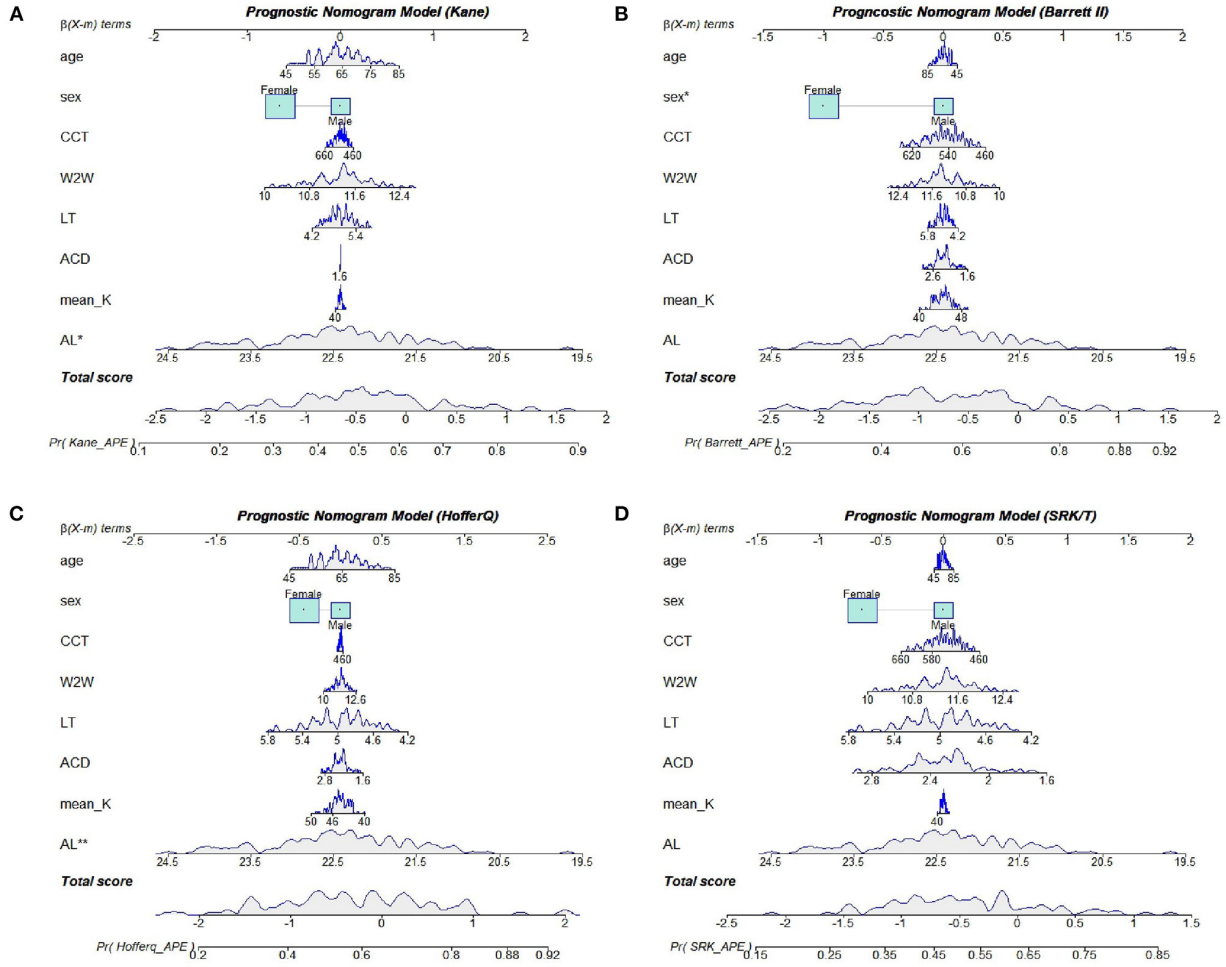
Nomogram models for the Kane **(A)**, Barrett II **(B)**, Hoffer Q **(C)**, and SRK/T **(D)** formula, respectively.

Based on the results of multivariate logistic regression analysis, we constructed four nomograms to predict refractive errors using different IOL power calculation formulas (Figure 4). All potential risk factors were included in the nomogram, and the effect-quantity of each factor was presented. The effect-quantity was used to show the impact of a potential risk factor on the event and then predict the probability of the event. The greatest effect-quantity in AL was displayed in almost all models, indicating its pivotal role in probability prediction. The AUC of these nomogram models was 0.690 (0.591, 0.789) for Kane, 0.701 (0.603, 0.799) for Barrett II, 0.708 (0.608, 0.808) for Hoffer Q, and 0.676 (0.575, 0.777) for SRKT formula, respectively. Hosmer–Lemeshow goodness-of-fit test showed that there was no significant difference between the actual and the predicted probability of refractive error events in each formula (*p*-value was 0.358 in the Kane, 0.724 in the Barrett II, 0.326 in the Hoffer Q, and 0.286 in the SRK/T, respectively), indicating a good predictive value.

## Discussion

To the best of our knowledge, this is the first work to evaluate the accuracy of new and traditional IOL calculation formulas and further use prognostic nomogram model to predict the refractive errors in eyes with PACG that underwent phaco-IOL-GSL. The results showed that the Kane and Barrett II formulas were comparable and outperformed the SRK/T and Hoffer Q in the total eyes with PACG. We also demonstrated that all four nomogram models had effective and reliable predictive power. We found that the AL had the greatest impact on the refractive outcomes, thus becoming a useful predictor to predict the risk of postoperative refractive error >0.50 D for patients with PACG.

Several investigations have similar results that phaco-IOL-GSL could effectively decline the peripheral anterior synechia (PAS) and IOP in patients with PACG, thus the cataract surgery could achieve ideal results with better visual acuity (4, 12). Among the emerging modern formulas, there is no consensus on which formula has the most excellent prediction in shallow anterior chamber eyes. The present work mainly focused on the patients with PACG who received phaco-IOL-GSL to control their IOP and evaluated the accuracy of IOL power calculation formulas. No significant difference was detected in the four formulas according to their absolute errors. However, Rhiu et al. (10) found that the SRK/T formula had lower MAE of 0.16 D compared with Hoffer Q and Holladay 2 formula. Gokce et al. (13) reported that Barrett II was more accurate with a smaller MedAE of 0.24 D than that of Hoffer Q, Haigis, and Olsen formulas in shallow anterior chamber eyes. Hipolito-Fernandes et al. (14) demonstrated that Kane formula had the lowest MedAE of 0.277 D and the highest percentage of eyes within ± 0.5 D among the six formulas including Barrett II, Hoffer Q, Haigis, SRK/T, Kane, and RBF 2.0 for the eyes with ACD <3.0 mm. The different results might be due to the inclusion disparity between previous studies and ours because patients who received antiglaucoma surgery previously were excluded in this work. Further investigations are needed to assess whether the operational treatment goniosynechialysis can significantly affect the IOL prediction. In our work, although without significance, the Kane formula produced the lowest MedAE of 0.49 D and the highest percentage of eyes within ± 0.50 D and ± 0.75 D, which was in accordance with the result of Hou et al. (15).

A primary angle-closure eye is characterized with a shorter AL, a smaller ACD, a smaller ACD/AL ratio, a thicker lens, and anterior rotation of ciliary processes (16), thereby leading to the inaccuracy of IOL power prediction. Hyperopic shift is commonly seen when an implanted IOL deviated from the planned position to a more posterior plane due to the deepening of the anterior chamber and a decrease of the AL (8, 17). However, significant myopic shift was noticed in this study when using the SRK/T and Hoffer Q formula, which was similar to the result of Kang et al. (5). The authors thought the instability of the implanted IOL due to the large capsular volume and loosened lens zonules in eyes with PACG contributed to this postoperative myopic shift (18). Taking the current evidence together, a certain anatomical change after cataract surgery makes this discrepancy in IOL power prediction. Comprehensively, Kane was indicated to be excellent in predicting the IOL power in eyes that underwent phaco-IOL-GSL, followed by the Barrett II. In addition, we found that the Hoffer Q was not as accurate as previous studies have reported (6, 19).

The prognostic factors incorporated in our nomograms were clinically accessible and economical. Our nomograms showed that the AL value played a pivotal role in the refractive error prediction, which was to say a shorter AL would lead to a larger risk of having postoperative refractive error >0.50 D. A previous study has reported that decreased AL could result in hyperopic shift in eyes with PACG after cataract extraction, which was in accordance with our results (17). In another study, Kang et al. (5) did not find any significant association between the extents of inaccuracy of IOL power calculation and preoperative anterior segment biometry such as ACD, AL, and LT. But they confirmed that hyperopic and myopic shifting was becoming more and more common in patients with PACG after cataract surgery, and appropriate management should be conducted in such patients. Several studies have showed that age might impact IOL prediction error of the SRK/T formula after cataract surgery (20, 21). It has been reported that older age was associated with greater postoperative refractive error. One possible reason is that the lens becomes more opaque and thicker when getting older, thus increasing the risk of glaucoma and affecting preoperative measurement of ocular biometrics. Moreover, age has been reported to affect the morphology of the Schlemm's canal (SC) and trabecular meshwork (TM) as well as the anterior chamber depth measurement, especially in patients with PACG (22, 23). However, age showed little effect-quantity and was not indicated as a significant predictor in the final nomogram models.

It should be noted that this study has several limitations. Firstly, the nomogram may have limited predictive power because of the relatively small sample size, but the AUC value and Hosmer–Lemeshow goodness-of-fit test have revealed good performance of our prognostic nomogram. Secondly, it is difficult to control the selection bias produced in this retrospective study, and the results may not be as persuasive as prospective studies. We have tried to control the bias through strict inclusion and exclusion criteria in a single race (24). Thirdly, some critical predictive factors, such as choroidal thickness and exact IOP data, were unavailable in our dataset, since maybe we have missed some of the important variables. However, the AL and CCT are thought to be related to the change of IOP, and studying only the ocular biometric variables could avoid the problem of collinearity (25, 26). Finally, the model accuracy has not been estimated with external validation based on other populations, and the AUC value of our nomograms were not relatively high.

In summary, the Kane and Barrett II formulas provided comparable outcomes, which achieved satisfying performance in the eyes with PACG that underwent phaco-IOL-GSL. Myopic outcomes could be seen in the Hoffer Q and SRK/T formulas in this kind of patients. Nomogram models indicated that the preoperative biometric parameter AL is a useful predictor to predict the probability of refractive error exceeding 0.50 D.

## Data Availability Statement

The raw data supporting the conclusions of this article will be made available by the authors, without undue reservation.

## Ethics Statement

The studies involving human participants were reviewed and approved by the Ethics Committee for Human Medical Research at the Joint Shantou International Eye Center of Shantou University and the Chinese University of Hong Kong (No. 2021JSIEC07015). Written informed consent for participation was not required for this study in accordance with the national legislation and the institutional requirements. Written informed consent was not obtained from the individual(s) for the publication of any potentially identifiable images or data included in this article.

## Author Contributions

YL was responsible for conceptualization, conducting the search, methodology, visualization, data curation, and writing the manuscript. CG was responsible for writing the manuscript, original draft preparation, conducting the search, resources, and investigation. LJ, YH, and RZ were responsible for conducting the search, resources, and investigation. CH was responsible for supervision and project administration. KQ and MZ were responsible for supervision, project administration, and reviewing the manuscript. All authors contributed to the article and approved the submitted version.

## Funding

This work was supported by the Shantou Medical Health, Science and Technology Project Fund [Project code: No. 106, Shanfu Section (2019)], Shantou, Guangdong, China.

## Conflict of Interest

The authors declare that the research was conducted in the absence of any commercial or financial relationships that could be construed as a potential conflict of interest.

## Publisher's Note

All claims expressed in this article are solely those of the authors and do not necessarily represent those of their affiliated organizations, or those of the publisher, the editors and the reviewers. Any product that may be evaluated in this article, or claim that may be made by its manufacturer, is not guaranteed or endorsed by the publisher.

## Footnotes

^1^ http://ocusoft.de/ulib/
