# Supplementary material for: Development and Evaluation of the Prognostic Nomogram to Predict Refractive Error in Patients With Primary Angle-Closure Glaucoma Who Underwent Cataract Surgery Combined With Goniosynechialysis
Source: Front Med (Lausanne). 2021 Dec 15;8:749903. doi: 10.3389/fmed.2021.749903 (PMC8714900; doi:10.3389/fmed.2021.749903)
Supplement: Supplementary file 1 [file Data_Sheet_1.PDF]

Supplementery material-1:source code for data analysis and nomogram construction

Yuancun Li

2021/11/5

1. Clear all environment variables

```
rm(list = ls())
```

2. Library packages

```
library(magrittr)
library(tidyverse)#for data processing
library(rms)#nomogram model construction and plot calibration curve
library(regplot)# plot the nomogram
library(pROC)#plot ROC curve
library(ResourceSelection)#Hosmer-Lemeshow test
```

3. Read data

```
df <- read.csv("data.csv",header=T)
df$age <- as.numeric(df$age)
str(df)
```

```
## 'data.frame':    111 obs. of  21 variables:
##  $ Pat_ID          : int  1141858 258705 1357044 1243958 1343516 1330534 1292779 940909 1281322 114[illegible]
##  $ sex             : chr  "Male" "Female" "Male" "Female" ...
##  $ age             : num  62 60 70 71 56 57 61 62 64 57 ...
##  $ eye             : chr  "OD" "OD" "OS" "OS" ...
##  $ device          : chr  "OA2000" "OA2000" "OA2000" "OA2000" ...
##  $ AL              : num  22.8 23.6 23.8 22.7 22.8 ...
##  $ K1              : num  44.6 42.2 43.5 43.8 44.9 ...
##  $ K2              : num  44.8 42.4 44.6 44.6 46.9 ...
##  $ K_mean          : num  44.7 42.3 44 44.2 45.9 ...
##  $ cyl             : num  -0.18 -0.212 -1.092 -0.869 -1.995 ...
##  $ ACD             : num  2.9 2.9 2.75 2.73 2.72 2.69 2.68 2.67 2.64 2.57 ...
##  $ LT              : num  4.82 4.33 5.22 4.45 4.63 4.69 4.51 4.75 5.06 4.96 ...
```

```
##  $ CCT               : num  476 593 534 501 585 501 643 511 490 526 ...
##  $ W2W               : num  11.9 11.9 12.1 11.5 12.1 ...
##  $ power_of_IOL      : num  23 22 20 24 21 22 27 20 23 26 ...
##  $ type_of_IOL       : chr  "970C" "920H" "920H" "970C" ...
##  $ actual_SE_diopter: num  -0.75 -0.375 -2.125 -2.25 -0.875 ...
##  $ srkt_reserved    : num  -0.72 -0.4 -0.72 -0.7 -0.62 -0.52 -1.44 -0.57 -0.28 -
0.32 ...
##  $ barrett_reserved : num  -0.71 -0.39 -0.74 -0.89 -0.7 -0.56 -1.62 -0.62 -0.36 -
0.09 ...
##  $ hofferq_reserved : num  -0.87 -0.07 -0.82 -0.71 -0.8 -0.5 -1.53 -0.51 -0.22 0.02 ...
##  $ kane_reserved    : num  -0.7 -0.4 -0.73 -0.94 -0.96 -0.82 -0.51 -0.73 -0.18 -
0.18 ...
```

As we can see,the data contains 111 objects and 21 variables.

4. Define events

```
data <- df %>%
  mutate(SRKT=actual_SE_diopter-srkt_reserved,
         Barrett=actual_SE_diopter-barrett_reserved,
         Hofferq=actual_SE_diopter-hofferq_reserved,
         kane=actual_SE_diopter-kane_reserved) %>%
  mutate(SRK_APE=ifelse(SRKT>=0.5|SRKT<=-0.5,1,0),
         Barrett_APE=ifelse(Barrett>=0.5|Barrett<=-0.5,1,0),
         Hofferq_APE=ifelse(Hofferq>=0.5|Hofferq<=-0.5,1,0),
         Kane_APE=ifelse(kane>=0.5|kane<=-0.5,1,0))

data$SRK_APE <- factor(data$SRK_APE,labels = c(0,1),levels=c(0,1))
data$Barrett_APE <- factor(data$Barrett_APE,labels = c(0,1),levels=c(0,1))
data$Hofferq_APE <- factor(data$Hofferq_APE,labels = c(0,1),levels=c(0,1))
data$Kane_APE <- factor(data$Kane_APE,labels = c(0,1),levels=c(0,1))
summary(data[,26:29])
```

```
##  SRK_APE Barrett_APE Hofferq_APE Kane_APE
##  0:54    0:49        0:48        0:55
##  1:57    1:62        1:63        1:56
```

Event is defined as absolute predictive error (APE) larger than or equal to 0.5 diopters, positive events are recorded as 1 and negative events are recorded as 0. As we can see, the number of positive event was 57,62,63,56 for SRKT, Barrett II, Hoffer Q and Kane formula, respectively.

5. logistic regression analysis and nomogram construction

The strategy of model construction are not data-driven due to the small sample size, but mostly based on clinical expertise.

5.1 SRKT formula

5.11 Univeriate logistic regression

```
AL <- glm(SRK_APE~ AL,family=binomial(link='logit'),data=data)
p <- summary(AL)
p$coefficients # p-value for coefficients
```

```
##               Estimate Std. Error   z value   Pr(>|z|)
## (Intercept) 10.7786801   5.237873  2.057835 0.03960594
## AL          -0.4781892   0.233352 -2.049219 0.04044074
```

```
exp(coef(AL)) #odds ratio
```

```
##  (Intercept)           AL
## 4.798675e+04 6.199049e-01
```

```
exp(confint(AL)) # 95% confidence interval
```

```
##                 2.5 %       97.5 %
## (Intercept) 2.2346698 2.162546e+09
## AL          0.3845787 9.667992e-01
```

```
K_mean <- glm(SRK_APE~ K_mean,family=binomial(link='logit'),data=data)
p <- summary(K_mean)
p$coefficients # p-value for coefficients
```

```
##               Estimate Std. Error   z value  Pr(>|z|)
## (Intercept) -7.1727431  5.5764587 -1.286254 0.1983544
## K_mean       0.1620957  0.1250261  1.296495 0.1948049
```

```
exp(coef(K_mean)) #odds ratio
```

```
##  (Intercept)       K_mean
## 0.0007672153 1.1759728198
```

```
exp(confint(K_mean)) # 95% confidence interval
```

```
##                    2.5 %    97.5 %
## (Intercept) 9.700135e-09 36.319729
## K_mean      9.238536e-01  1.514432
```

```
ACD <- glm(SRK_APE~ ACD,family=binomial(link='logit'),data=data)
p <- summary(ACD)
p$coefficients # p-value for coefficients
```

```
##               Estimate Std. Error    z value  Pr(>|z|)
## (Intercept)  1.3107977  1.8999709  0.6899041 0.4902545
## ACD         -0.5432874  0.8170631 -0.6649272 0.5060971
```

```r
exp(coef(ACD)) #odds ratio
```

```
## (Intercept)         ACD
##   3.7091314   0.5808357
```

```r
exp(confint(ACD)) # 95% confidence interval
```

```
##                  2.5 %     97.5 %
## (Intercept) 0.09095658 167.997601
## ACD         0.11280677   2.863585
```

```r
LT <- glm(SRK_APE~ LT,family=binomial(link='logit'),data=data)
p <- summary(LT)
p$coefficients # p-value for coefficients
```

```
##              Estimate Std. Error    z value  Pr(>|z|)
## (Intercept)  2.806506  3.0147689  0.9309189 0.3518955
## LT          -0.553453  0.6049502 -0.9148737 0.3602580
```

```r
exp(coef(LT)) #odds ratio
```

```
## (Intercept)          LT
##   16.551977    0.574961
```

```r
exp(confint(LT)) # 95% confidence interval
```

```
##                  2.5 %      97.5 %
## (Intercept) 0.04669893 6920.172879
## LT          0.17123204    1.867858
```

```r
W2W <- glm(SRK_APE~ W2W,family=binomial(link='logit'),data=data)
p <- summary(W2W)
p$coefficients # p-value for coefficients
```

```
##              Estimate Std. Error    z value  Pr(>|z|)
## (Intercept)  1.655227  4.5456506  0.3641343 0.7157577
## W2W         -0.141134  0.4003082 -0.3525635 0.7244157
```

```r
exp(coef(W2W)) #odds ratio
```

```
## (Intercept)         W2W
##   5.2342705   0.8683729
```

```r
exp(confint(W2W)) # 95% confidence interval
```

```
##                   2.5 %       97.5 %
## (Intercept) 0.000694441 44697.178928
## W2W         0.391380492     1.906364
```

```r
CCT <- glm(SRK_APE~ CCT,family=binomial(link='logit'),data=data)
p <- summary(CCT)
p$coefficients # p-value for coefficients
```

```
##                 Estimate Std. Error    z value  Pr(>|z|)
## (Intercept)  1.055281342 2.80824526  0.3757796 0.7070807
## CCT         -0.001811507 0.00506909 -0.3573634 0.7208198
```

```r
exp(coef(CCT)) #odds ratio
```

```
## (Intercept)         CCT
##   2.8727833   0.9981901
```

```r
exp(confint(CCT)) # 95% confidence interval
```

```
##                  2.5 %     97.5 %
## (Intercept) 0.01156622 755.696744
## CCT         0.98820085   1.008181
```

```r
sex <- glm(SRK_APE~ sex,family=binomial(link='logit'),data=data)
p <- summary(sex)
p$coefficients # p-value for coefficients
```

```
##                Estimate Std. Error    z value  Pr(>|z|)
## (Intercept) -0.05129329  0.2265299 -0.2264306 0.8208665
## sexMale      0.35667494  0.4187714  0.8517175 0.3943709
```

```r
exp(coef(sex)) #odds ratio
```

```
## (Intercept)     sexMale
##    0.950000    1.428571
```

```r
exp(confint(sex)) # 95% confidence interval
```

```
##                 2.5 %   97.5 %
## (Intercept) 0.6077654 1.482432
## sexMale     0.6314683 3.290080
```

```r
age <- glm(SRK_APE~ age,family=binomial(link='logit'),data=data)
p <- summary(age)
p$coefficients # p-value for coefficients
```

```
##                 Estimate Std. Error    z value  Pr(>|z|)
## (Intercept)  0.334836732 1.53156286  0.2186242 0.8269428
## age         -0.004372609 0.02366654 -0.1847591 0.8534180
```

```
exp(coef(age)) #odds ratio
```

```
## (Intercept)         age
##   1.3977122   0.9956369
```

```
exp(confint(age)) # 95% confidence interval
```

```
##                  2.5 %    97.5 %
## (Intercept) 0.06860368 29.056667
## age         0.95005457  1.043138
```

5.12 Multivariate logistic regression

```
formula=SRK_APE~ AL+K_mean+ACD+LT+W2W+CCT+sex+age
fit1 <- glm(formula=formula,family=binomial(link='logit'),data=data)
p <- summary(fit1)
p$coefficients # p-value for coefficients
```

```
##                 Estimate   Std. Error     z value   Pr(>|z|)
## (Intercept) 18.010732496 14.682344141  1.22669325 0.21993790
## AL          -0.668264730  0.350282447 -1.90778823 0.05641859
## K_mean       0.010259076  0.172646191  0.05942254 0.95261556
## ACD         -1.176462890  0.992797618 -1.18499770 0.23601836
## LT          -0.921432801  0.703271185 -1.31020980 0.19012487
## W2W          0.456340923  0.498377972  0.91565227 0.35984930
## CCT         -0.003147479  0.005485509 -0.57378078 0.56611616
## sexMale      0.651783080  0.471761367  1.38159486 0.16709613
## age          0.003938863  0.026530580  0.14846502 0.88197579
```

```
exp(coef(fit1)) #odds ratio
```

```
##  (Intercept)           AL       K_mean          ACD            LT           W2W
## 6.636846e+07 5.125973e-01 1.010312e+00 3.083675e-01 3.979485e-01 1.578288e+00
##          CCT      sexMale          age
## 9.968575e-01 1.918959e+00 1.003947e+00
```

```
exp(confint(fit1)) # 95% confidence interval
```

```
##                    2.5 %       97.5 %
## (Intercept) 2.843855e-05 5.068515e+20
## AL          2.498301e-01 1.001210e+00
## K_mean      7.163190e-01 1.420374e+00
## ACD         4.080501e-02 2.084291e+00
## LT          9.598284e-02 1.549356e+00
## W2W         5.989871e-01 4.297785e+00
## CCT         9.860568e-01 1.007656e+00
## sexMale     7.713330e-01 4.963772e+00
## age         9.529271e-01 1.058374e+00
```

5.13 nomogram construction

```
dd <- datadist(data)
options(datadist="dd")
formula=SRK_APE~ age+sex+CCT+W2W+LT+ACD+K_mean+AL
fit <- lrm(formula = formula,data=data,x=T,y=T)
nom <- nomogram(fit,
         fun=function(x)1/(1+exp(-x)),
         fun.at = c(0.001,0.01,0.05,seq(0.1,0.9,by=0.1),0.95,0.99,0.999),
         lp=F,
         funlabel = "Pr(SRK/T)")
plot(nom)
```

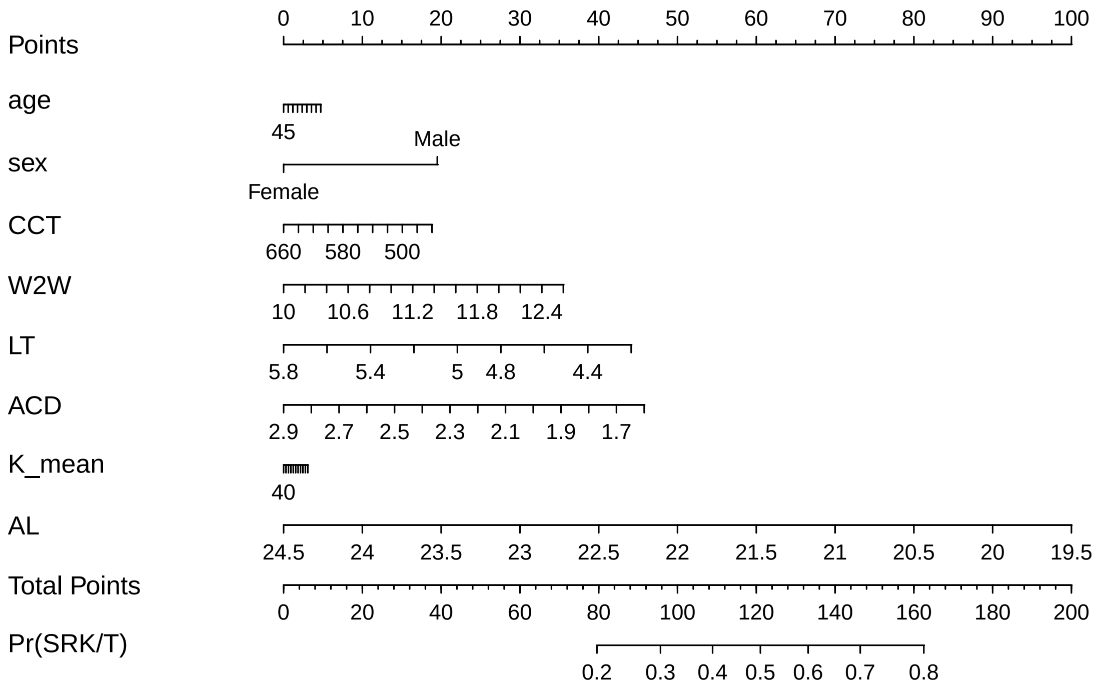

5.14 Accuracy of the model

We plot a ROC curve here, and the area under the ROC curve was used to represent the accuracy of nomogram model.

```
fit1 <- glm(formula = formula,family=binomial(link='logit'),data=data)
pre = predict(fit1,type="response")
modelroc <- pROC::roc(data$SRK_APE,pre,
```

```
                          ci=T, # arguments for ci
                          boot.n=300,
                          ci.alpha=0.95,
                          stratified=T)
plot(modelroc,
     print.auc=T,
     auc.polygon=T,
     grid=c(0.1,0.2),
     grid.col=c("#FFF0F5","#FFC0CB"),
     max.auc.polygon=T,
     auc.polygon.col="#CCCCFF",
     print.thres=F)
```

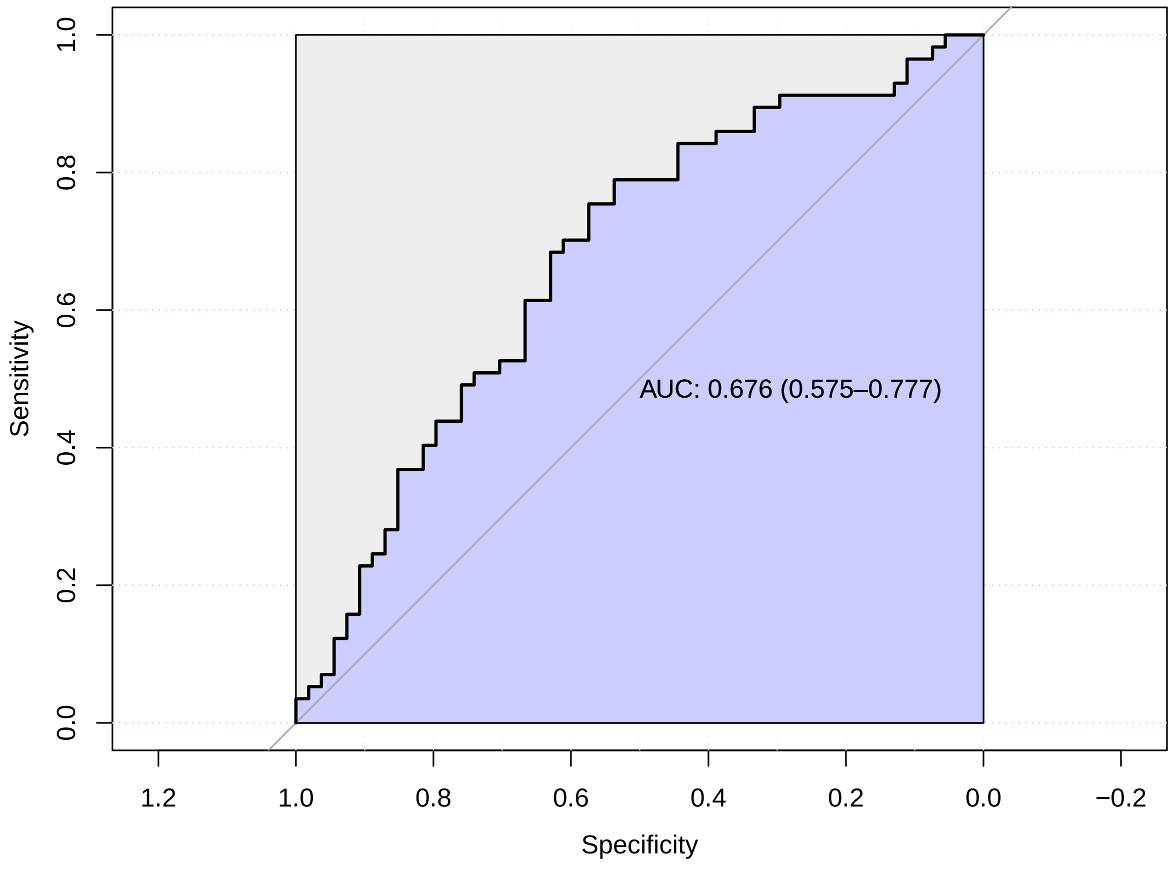

5.15 Calibration plot and Hosmer-Lemeshow test

Nomogram model was validated using calibration curve with bootstrap method, and Hosmer-Lemeshow goodness-of-fit test was conducted.

```
nom <- lrm(formula = formula,data=data,x=T,y=T)
cal <- calibrate(nom,method="boot",B=100)
par(mar=c(8,4,1,2))
plot(cal)
```

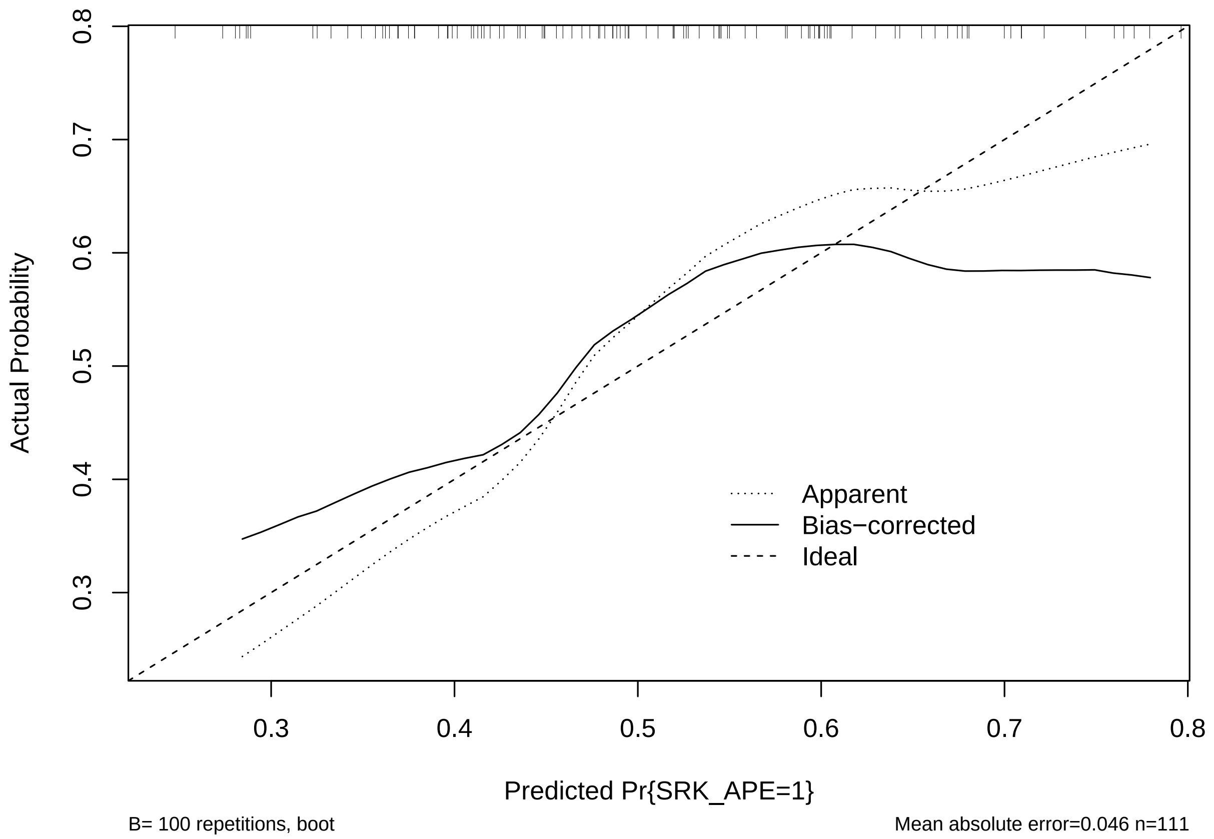


```
##
## n=111   Mean absolute error=0.046   Mean squared error=0.00391
## 0.9 Quantile of absolute error=0.099
```

```
# HL-test
h.test <- hoslem.test(fit1$y, fitted(fit1), g=10)
h.test
```

```
##
##  Hosmer and Lemeshow goodness of fit (GOF) test
##
## data:  fit1$y, fitted(fit1)
## X-squared = 9.7145, df = 8, p-value = 0.2856
```

5.2 HofferQ formula

5.21 Univeriate logistic regression

```
AL <- glm(Hofferq_APE~ AL,family=binomial(link='logit'),data=data)
p <- summary(AL)
p$coefficients # p-value for coefficients
```

```
##               Estimate Std. Error   z value    Pr(>|z|)
## (Intercept) 17.4994179  5.7178864  3.060470 0.002209901
## AL          -0.7671226  0.2541616 -3.018247 0.002542414
```

```r
exp(coef(AL)) #odds ratio
```

```
##  (Intercept)           AL
## 3.980161e+07 4.643472e-01
```

```r
exp(confint(AL)) # 95% confidence interval
```

```
##                  2.5 %       97.5 %
## (Intercept) 893.242763 5.691749e+12
## AL            0.274003 7.474975e-01
```

```r
K_mean <- glm(Hofferq_APE~ K_mean,family=binomial(link='logit'),data=data)
p <- summary(K_mean)
p$coefficients # p-value for coefficients
```

```
##               Estimate Std. Error   z value   Pr(>|z|)
## (Intercept) -9.6465031  5.7460113 -1.678817 0.09318766
## K_mean       0.2226235  0.1290064  1.725679 0.08440525
```

```r
exp(coef(K_mean)) #odds ratio
```

```
##  (Intercept)       K_mean
## 6.465125e-05 1.249350e+00
```

```r
exp(confint(K_mean)) # 95% confidence interval
```

```
##                    2.5 %   97.5 %
## (Intercept) 5.340486e-10 3.870282
## K_mean      9.761848e-01 1.625197
```

```r
ACD <- glm(Hofferq_APE~ ACD,family=binomial(link='logit'),data=data)
p <- summary(ACD)
p$coefficients # p-value for coefficients
```

```
##               Estimate Std. Error    z value  Pr(>|z|)
## (Intercept)  0.6137983  1.9050877  0.3221890 0.7473095
## ACD         -0.1477820  0.8191636 -0.1804059 0.8568339
```

```r
exp(coef(ACD)) #odds ratio
```

```
## (Intercept)         ACD
##   1.8474351   0.8626192
```

```r
exp(confint(ACD)) # 95% confidence interval
```

```
##                   2.5 %    97.5 %
## (Intercept) 0.04371373 82.390373
## ACD         0.16921301  4.331257
```

```r
LT <- glm(Hofferq_APE~ LT,family=binomial(link='logit'),data=data)
p <- summary(LT)
p$coefficients # p-value for coefficients
```

```
##                Estimate Std. Error   z value  Pr(>|z|)
## (Intercept)  4.4548566  3.0742227  1.449100 0.1473096
## LT          -0.8403076  0.6157907 -1.364599 0.1723790
```

```r
exp(coef(LT)) #odds ratio
```

```
## (Intercept)          LT
##  86.0438081   0.4315777
```

```r
exp(confint(LT)) # 95% confidence interval
```

```
##                  2.5 %      97.5 %
## (Intercept) 0.2251248 42231.99784
## LT          0.1248960     1.42236
```

```r
W2W <- glm(Hofferq_APE~ W2W,family=binomial(link='logit'),data=data)
p <- summary(W2W)
p$coefficients # p-value for coefficients
```

```
##                Estimate Std. Error   z value  Pr(>|z|)
## (Intercept)  6.8226040  4.7301264  1.442372 0.1491973
## W2W         -0.5769675  0.4159313 -1.387170 0.1653899
```

```r
exp(coef(W2W)) #odds ratio
```

```
## (Intercept)         W2W
## 918.3733820   0.5615988
```

```r
exp(confint(W2W)) # 95% confidence interval
```

```
##                  2.5 %       97.5 %
## (Intercept) 0.1034434 1.375874e+07
## W2W         0.2413556 1.250233e+00
```

```r
CCT <- glm(Hofferq_APE~ CCT,family=binomial(link='logit'),data=data)
p <- summary(CCT)
p$coefficients # p-value for coefficients
```

```
##                 Estimate  Std. Error    z value  Pr(>|z|)
## (Intercept) -0.609870668 2.833605174 -0.2152278 0.8295897
## CCT          0.001595993 0.005118292  0.3118214 0.7551763
```

```r
exp(coef(CCT)) #odds ratio
```

```
## (Intercept)         CCT
##   0.5434211   1.0015973
```

```r
exp(confint(CCT)) # 95% confidence interval
```

```
##                   2.5 %     97.5 %
## (Intercept) 0.001967863 142.187703
## CCT         0.991592430   1.011838
```

```r
sex <- glm(Hofferq_APE~ sex,family=binomial(link='logit'),data=data)
p <- summary(sex)
p$coefficients # p-value for coefficients
```

```
##               Estimate Std. Error   z value  Pr(>|z|)
## (Intercept) 0.25782911  0.2283397 1.1291469 0.2588359
## sexMale     0.04755254  0.4197609 0.1132848 0.9098047
```

```r
exp(coef(sex)) #odds ratio
```

```
## (Intercept)     sexMale
##    1.294118    1.048701
```

```r
exp(confint(sex)) # 95% confidence interval
```

```
##                 2.5 %   97.5 %
## (Intercept) 0.8292036 2.037172
## sexMale     0.4619341 2.416328
```

```r
age <- glm(Hofferq_APE~ age,family=binomial(link='logit'),data=data)
p <- summary(age)
p$coefficients # p-value for coefficients
```

```
##                Estimate Std. Error    z value  Pr(>|z|)
## (Intercept) -0.38509946 1.54592318 -0.2491065 0.8032784
## age          0.01024014 0.02392449  0.4280192 0.6686372
```

```r
exp(coef(age)) #odds ratio
```

```
## (Intercept)         age
##    0.680383    1.010293
```

```r
exp(confint(age)) # 95% confidence interval
```

```
##                   2.5 %    97.5 %
## (Intercept) 0.03176784 14.244885
## age         0.96398323  1.059512
```

5.22 Multivariate logistic regression

```r
formula=Hofferq_APE~ AL+K_mean+ACD+LT+W2W+CCT+sex+age
fit1 <- glm(formula,family=binomial(link='logit'),data=data)
p <- summary(fit1)
p$coefficients # p-value for coefficients
```

```
##                  Estimate   Std. Error     z value    Pr(>|z|)
## (Intercept) 28.0449284745 15.760681666  1.77942357 0.075170345
## AL          -0.9993891638  0.382732068 -2.61119788 0.009022567
## K_mean      -0.0643890306  0.183589160 -0.35072349 0.725795802
## ACD         -0.3806644686  1.010171326 -0.37683159 0.706298762
## LT          -1.0547113022  0.724682564 -1.45541145 0.145555388
## W2W          0.1537366563  0.504449486  0.30476125 0.760547996
## CCT         -0.0004129952  0.005728484 -0.07209503 0.942526281
## sexMale      0.4347387215  0.486316004  0.89394286 0.371352462
## age          0.0316510496  0.027860606  1.13605029 0.255935519
```

```r
exp(coef(fit1)) #odds ratio
```

```
##  (Intercept)           AL       K_mean          ACD           LT          W2W
## 1.512717e+12 3.681042e-01 9.376402e-01 6.834072e-01 3.482930e-01 1.166184e+00
##          CCT      sexMale          age
## 9.995871e-01 1.544559e+00 1.032157e+00
```

```r
exp(confint(fit1)) # 95% confidence interval
```

```
##                  2.5 %       97.5 %
## (Intercept) 0.13395147 1.530978e+26
## AL          0.16571782 7.523242e-01
## K_mean      0.64809924 1.339545e+00
## ACD         0.08940615 4.908592e+00
## LT          0.08042080 1.413328e+00
## W2W         0.43002573 3.167528e+00
## CCT         0.98839432 1.011012e+00
## sexMale     0.60343914 4.116591e+00
## age         0.97838259 1.092425e+00
```

5.23 nomogram construction

```
dd <- datadist(data)
options(datadist="dd")
formula=Hofferq_APE~ age+sex+CCT+W2W+LT+ACD+K_mean+AL
fit <- lrm(formula = formula,data=data,x=T,y=T)
nom <- nomogram(fit,
         fun=function(x)1/(1+exp(-x)),
         fun.at = c(0.001,0.01,0.05,seq(0.1,0.9,by=0.1),0.95,0.99,0.999),
         lp=F,
         funlabel = "Pr(Hoffer Q)")
plot(nom)
```

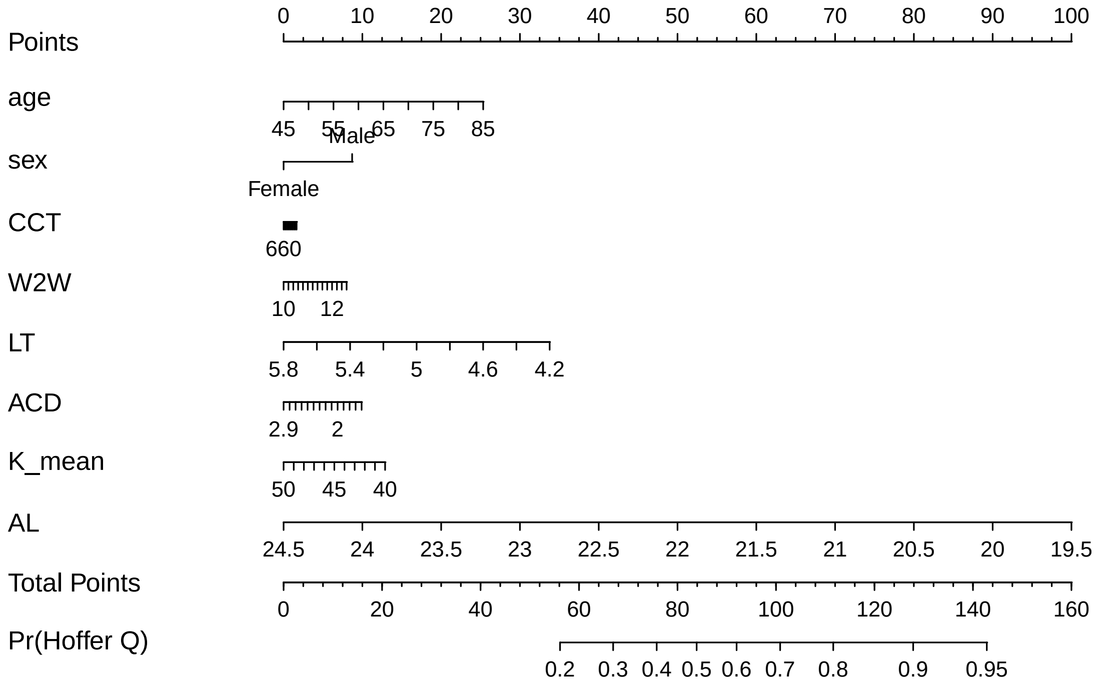

5.24 Accuracy of the model

We plot a ROC curve here, and the area under the ROC curve was used to represent the accuracy of nomogram model.

```
fit1 <- glm(formula = formula,family=binomial(link='logit'),data=data)
pre = predict(fit1,type="response")
modelroc <- pROC::roc(data$Hofferq_APE,pre,
                      ci=T, # arguments for ci
                      boot.n=300,
```

```
                      ci.alpha=0.95,
                      stratified=T)
plot(modelroc,
     print.auc=T,
     auc.polygon=T,
     grid=c(0.1,0.2),
     grid.col=c("#FFF0F5","#FFC0CB"),
     max.auc.polygon=T,
     auc.polygon.col="#CCCCFF",
     print.thres=F)
```

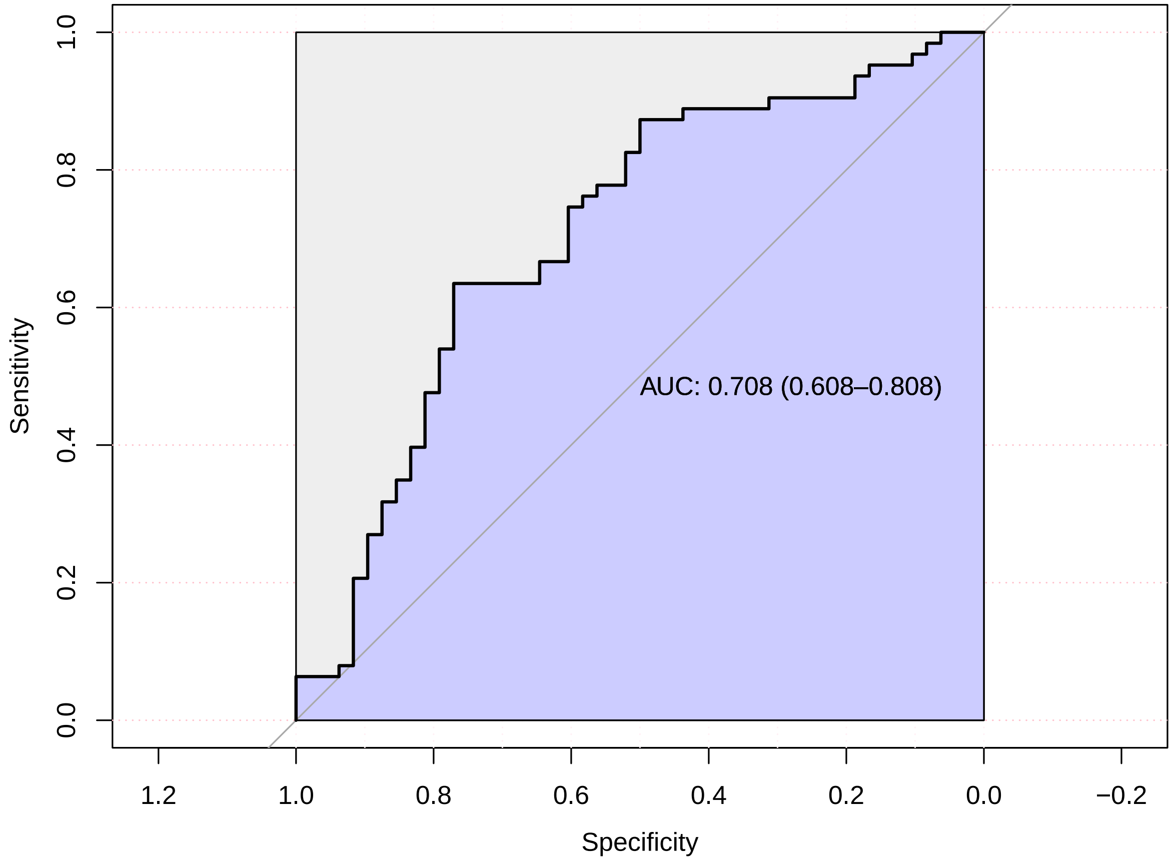

5.25 Calibration plot and Hosmer-Lemeshow test

Nomogram model was validated using calibration curve with bootstrap method, and Hosmer-Lemeshow goodness-of-fit test was conducted.

```
nom <- lrm(formula = formula,data=data,x=T,y=T)
cal <- calibrate(nom,method="boot",B=100)
par(mar=c(8,4,1,2))
plot(cal)
```

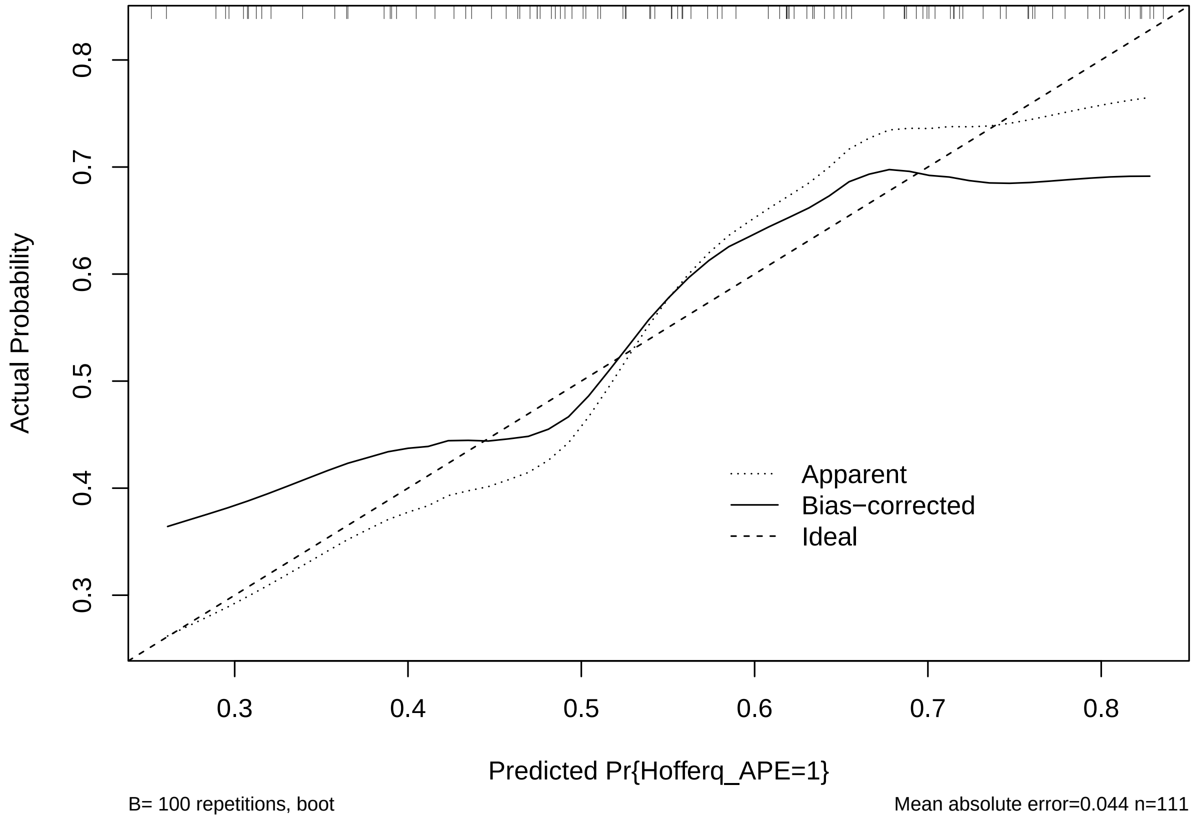


```
##
## n=111   Mean absolute error=0.044   Mean squared error=0.00313
## 0.9 Quantile of absolute error=0.092
```

```
# HL-test
h.test <- hoslem.test(fit1$y, fitted(fit1), g=10)
h.test
```

```
##
##  Hosmer and Lemeshow goodness of fit (GOF) test
##
## data:  fit1$y, fitted(fit1)
## X-squared = 9.201, df = 8, p-value = 0.3256
```

5.3 Kane formula

5.31 Univeriate logistic regression

```
AL <- glm(Kane_APE~ AL,family=binomial(link='logit'),data=data)
p <- summary(AL)
p$coefficients # p-value for coefficients
```

```
##                Estimate Std. Error   z value   Pr(>|z|)
## (Intercept) 12.2207664   5.323779  2.295506 0.02170416
## AL          -0.5441704   0.237249 -2.293668 0.02180960
```

```
exp(coef(AL)) #odds ratio
```

```
##  (Intercept)           AL
## 2.029605e+05 5.803230e-01
```

```
exp(confint(AL)) # 95% confidence interval
```

```
##                 2.5 %       97.5 %
## (Intercept) 8.4017604 1.138980e+10
## AL          0.3564476 9.098906e-01
```

```
K_mean <- glm(Kane_APE~ K_mean,family=binomial(link='logit'),data=data)
p <- summary(K_mean)
p$coefficients # p-value for coefficients
```

```
##                Estimate Std. Error   z value  Pr(>|z|)
## (Intercept) -8.5062655  5.6249781 -1.512231 0.1304751
## K_mean       0.1911862  0.1261008  1.516138 0.1294846
```

```
exp(coef(K_mean)) #odds ratio
```

```
##  (Intercept)       K_mean
## 0.0002021975 1.2106848298
```

```
exp(confint(K_mean)) # 95% confidence interval
```

```
##                    2.5 %    97.5 %
## (Intercept) 2.225516e-09 10.058251
## K_mean      9.500703e-01  1.563911
```

```
ACD <- glm(Kane_APE~ ACD,family=binomial(link='logit'),data=data)
p <- summary(ACD)
p$coefficients # p-value for coefficients
```

```
##                Estimate Std. Error    z value  Pr(>|z|)
## (Intercept)  0.4768351  1.8871411  0.2526759 0.8005187
## ACD         -0.1983807  0.8117898 -0.2443745 0.8069408
```

```
exp(coef(ACD)) #odds ratio
```

```
## (Intercept)         ACD
##   1.6109677   0.8200576
```

```r
exp(confint(ACD)) # 95% confidence interval
```

```
##                  2.5 %    97.5 %
## (Intercept) 0.03917992 68.814404
## ACD         0.16316351  4.056889
```

```r
LT <- glm(Kane_APE~ LT,family=binomial(link='logit'),data=data)
p <- summary(LT)
p$coefficients # p-value for coefficients
```

```
##               Estimate Std. Error    z value  Pr(>|z|)
## (Intercept) -2.3268456  3.0068196 -0.7738561 0.4390159
## LT           0.4715846  0.6035626  0.7813350 0.4346055
```

```r
exp(coef(LT)) #odds ratio
```

```
## (Intercept)          LT
##  0.09760314  1.60253154
```

```r
exp(confint(LT)) # 95% confidence interval
```

```
##                    2.5 %    97.5 %
## (Intercept) 0.0002399305 34.447674
## LT          0.4937329152  5.356087
```

```r
W2W <- glm(Kane_APE~ W2W,family=binomial(link='logit'),data=data)
p <- summary(W2W)
p$coefficients # p-value for coefficients
```

```
##                Estimate Std. Error    z value  Pr(>|z|)
## (Intercept) -0.63972778  4.5363092 -0.1410238 0.8878511
## W2W          0.05797958  0.3995218  0.1451225 0.8846142
```

```r
exp(coef(W2W)) #odds ratio
```

```
## (Intercept)         W2W
##    0.527436    1.059693
```

```r
exp(confint(W2W)) # 95% confidence interval
```

```
##                    2.5 %      97.5 %
## (Intercept) 6.548594e-05 4062.852059
## W2W         4.818592e-01    2.339813
```

```r
CCT <- glm(Kane_APE~ CCT,family=binomial(link='logit'),data=data)
p <- summary(CCT)
p$coefficients # p-value for coefficients
```

```
##                  Estimate  Std. Error    z value  Pr(>|z|)
## (Intercept)  0.4294536539 2.804165000  0.1531485 0.8782812
## CCT         -0.0007444502 0.005062177 -0.1470613 0.8830837
```

```r
exp(coef(CCT)) #odds ratio
```

```
## (Intercept)         CCT
##   1.5364179   0.9992558
```

```r
exp(confint(CCT)) # 95% confidence interval
```

```
##                   2.5 %     97.5 %
## (Intercept) 0.006131385 394.199131
## CCT         0.989297940   1.009272
```

```r
sex <- glm(Kane_APE~ sex,family=binomial(link='logit'),data=data)
p <- summary(sex)
p$coefficients # p-value for coefficients
```

```
##               Estimate Std. Error    z value  Pr(>|z|)
## (Intercept) -0.1026542  0.2267537 -0.4527122 0.6507560
## sexMale      0.4080358  0.4188925  0.9740824 0.3300157
```

```r
exp(coef(sex)) #odds ratio
```

```
## (Intercept)     sexMale
##    0.902439    1.503861
```

```r
exp(confint(sex)) # 95% confidence interval
```

```
##                 2.5 %   97.5 %
## (Intercept) 0.5765900 1.407644
## sexMale     0.6647561 3.465083
```

```r
age <- glm(Kane_APE~ age,family=binomial(link='logit'),data=data)
p <- summary(age)
p$coefficients # p-value for coefficients
```

```
##                Estimate Std. Error    z value  Pr(>|z|)
## (Intercept) -1.36975414 1.54483082 -0.8866693 0.3752569
## age          0.02161631 0.02388176  0.9051391 0.3653917
```

```r
exp(coef(age)) #odds ratio
```

```
## (Intercept)         age
##   0.2541694   1.0218516
```

```r
exp(confint(age)) # 95% confidence interval
```

```
##                  2.5 %   97.5 %
## (Intercept) 0.01154511 5.153074
## age         0.97541708 1.071895
```

5.32 Multivariate logistic regression

```r
formula=Kane_APE~ AL+K_mean+ACD+LT+W2W+CCT+sex+age
fit1 <- glm(formula,family=binomial(link='logit'),data=data)
p <- summary(fit1)
p$coefficients # p-value for coefficients
```

```
##                 Estimate   Std. Error      z value  Pr(>|z|)
## (Intercept)  9.316579596 15.116356630  0.616324411 0.5376804
## AL          -0.891115045  0.371049379 -2.401607699 0.0163232
## K_mean       0.011620096  0.179255021  0.064824380 0.9483138
## ACD          0.008194456  0.985692092  0.008313404 0.9933669
## LT           0.396980391  0.704850823  0.563211928 0.5732906
## W2W          0.607585335  0.507093989  1.198171046 0.2308504
## CCT         -0.001552011  0.005574835 -0.278395791 0.7807086
## sexMale      0.644403952  0.478467849  1.346807216 0.1780423
## age          0.030392682  0.027073398  1.122603152 0.2616061
```

```r
exp(coef(fit1)) #odds ratio
```

```
##  (Intercept)           AL       K_mean          ACD           LT          W2W
## 1.112088e+04 4.101981e-01 1.011688e+00 1.008228e+00 1.487327e+00 1.835993e+00
##          CCT      sexMale          age
## 9.984492e-01 1.904851e+00 1.030859e+00
```

```r
exp(confint(fit1)) # 95% confidence interval
```

```
##                    2.5 %       97.5 %
## (Intercept) 1.743281e-09 1.583148e+17
## AL          1.894842e-01 8.224017e-01
## K_mean      7.072955e-01 1.437834e+00
## ACD         1.426292e-01 7.111212e+00
## LT          3.755161e-01 6.104100e+00
## W2W         6.888932e-01 5.116805e+00
## CCT         9.874936e-01 1.009491e+00
## sexMale     7.555442e-01 4.993800e+00
## age         9.783311e-01 1.088934e+00
```

5.33 nomogram construction

```
dd <- datadist(data)
options(datadist="dd")
formula=Kane_APE~ age+sex+CCT+W2W+LT+ACD+K_mean+AL
fit <- lrm(formula = formula,data=data,x=T,y=T)
nom <- nomogram(fit,
         fun=function(x)1/(1+exp(-x)),
         fun.at = c(0.001,0.01,0.05,seq(0.1,0.9,by=0.1),0.95,0.99,0.999),
         lp=F,
         funlabel = "Pr(Kane)")
plot(nom)
```

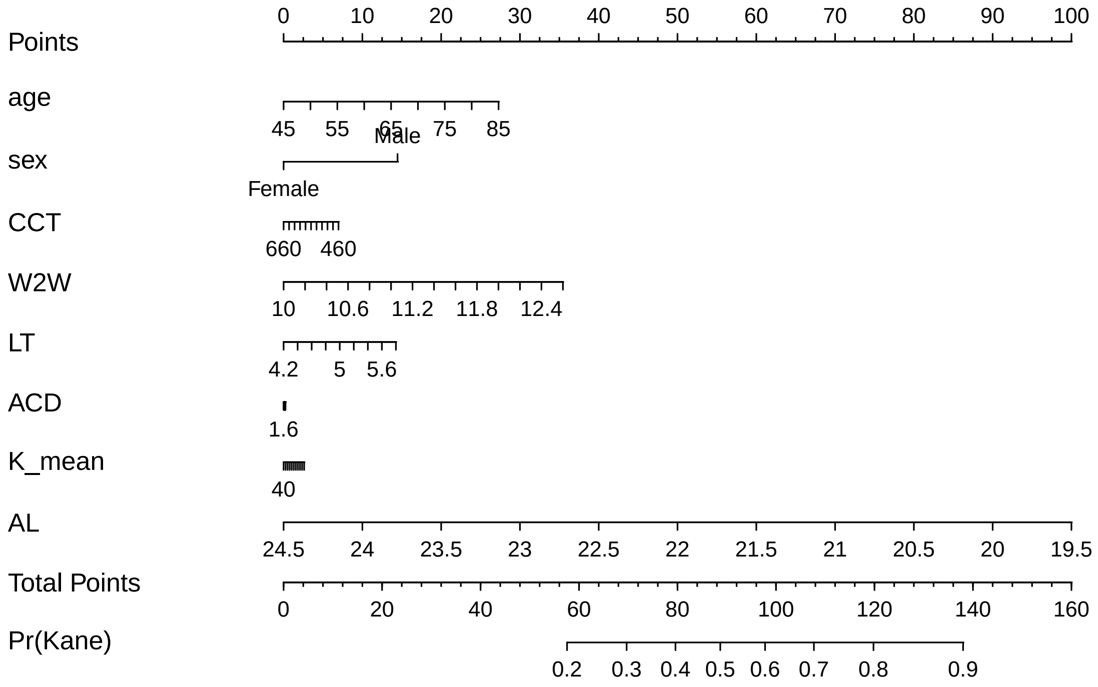

5.34 Accuracy of the model

We plot a ROC curve here, and the area under the ROC curve was used to represent the accuracy of nomogram model.

```
fit1 <- glm(formula = formula,family=binomial(link='logit'),data=data)
pre = predict(fit1,type="response")
modelroc <- pROC::roc(data$Kane_APE,pre,
                      ci=T, # arguments for ci
                      boot.n=300,
```

```
                       ci.alpha=0.95,
                       stratified=T)
plot(modelroc,
     print.auc=T,
     auc.polygon=T,
     grid=c(0.1,0.2),
     grid.col=c("#FFF0F5","#FFC0CB"),
     max.auc.polygon=T,
     auc.polygon.col="#CCCCFF",
     print.thres=F)
```

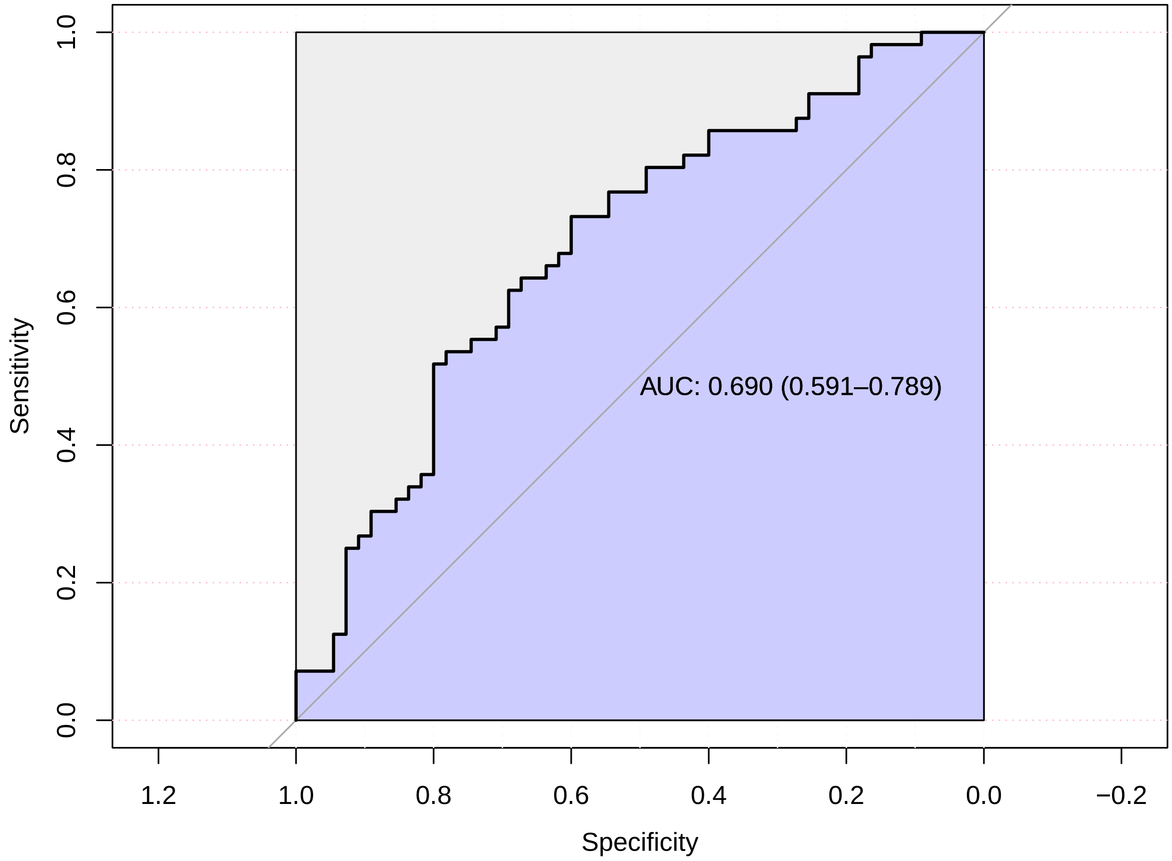

5.35 Calibration plot and Hosmer-Lemeshow test

Nomogram model was validated using calibration curve with bootstrap method, and Hosmer-Lemeshow goodness-of-fit test was conducted.

```
nom <- lrm(formula = formula,data=data,x=T,y=T)
cal <- calibrate(nom,method="boot",B=100)
par(mar=c(8,4,1,2))
plot(cal)
```

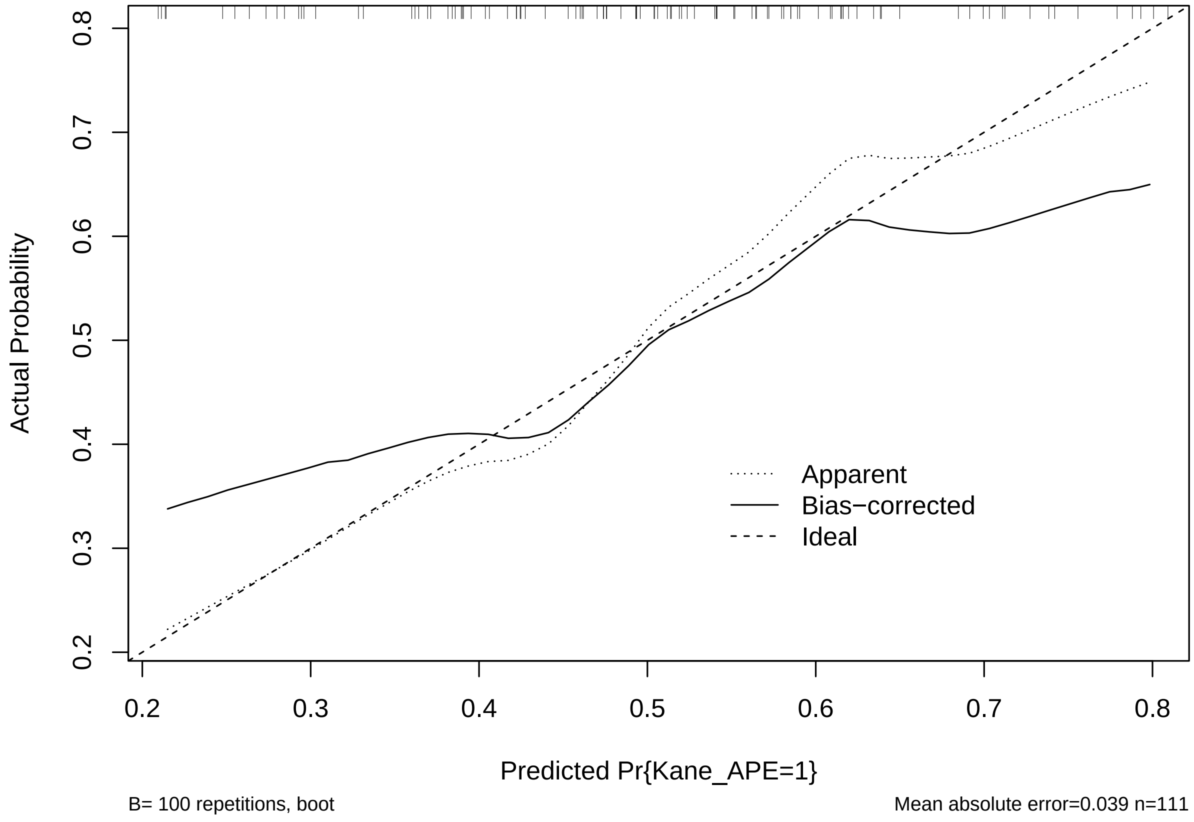


```
##
## n=111   Mean absolute error=0.039   Mean squared error=0.00324
## 0.9 Quantile of absolute error=0.107
```

```
# HL-test
h.test <- hoslem.test(fit1$y, fitted(fit1), g=10)
h.test
```

```
##
##  Hosmer and Lemeshow goodness of fit (GOF) test
##
## data:  fit1$y, fitted(fit1)
## X-squared = 8.8233, df = 8, p-value = 0.3574
```

5.4 Barrett formula

5.41 Univeriate logistic regression

```
AL <- glm(Barrett_APE~ AL,family=binomial(link='logit'),data=data)
p <- summary(AL)
p$coefficients # p-value for coefficients
```

```
##               Estimate Std. Error   z value    Pr(>|z|)
## (Intercept) 15.278851  5.5431083  2.756369 0.005844706
## AL          -0.670102  0.2465238 -2.718204 0.006563739
```

```
exp(coef(AL)) #odds ratio
```

```
##  (Intercept)           AL
## 4.320366e+06 5.116564e-01
```

```
exp(confint(AL)) # 95% confidence interval
```

```
##                    2.5 %       97.5 %
## (Intercept) 127.2703340 4.067974e+11
## AL            0.3074941 8.139420e-01
```

```
K_mean <- glm(Barrett_APE~ K_mean,family=binomial(link='logit'),data=data)
p <- summary(K_mean)
p$coefficients # p-value for coefficients
```

```
##                 Estimate Std. Error   z value   Pr(>|z|)
## (Intercept) -10.8242967  5.7911389 -1.869114 0.06160701
## K_mean        0.2482327  0.1300154  1.909256 0.05622909
```

```
exp(coef(K_mean)) #odds ratio
```

```
##  (Intercept)       K_mean
## 1.990984e-05 1.281758e+00
```

```
exp(confint(K_mean)) # 95% confidence interval
```

```
##                    2.5 %   97.5 %
## (Intercept) 1.451203e-10 1.251838
## K_mean      1.000385e+00 1.671986
```

```
ACD <- glm(Barrett_APE~ ACD,family=binomial(link='logit'),data=data)
p <- summary(ACD)
p$coefficients # p-value for coefficients
```

```
##               Estimate Std. Error    z value  Pr(>|z|)
## (Intercept)  1.0193191   1.906234  0.5347294 0.5928370
## ACD         -0.3388307   0.819282 -0.4135703 0.6791888
```

```
exp(coef(ACD)) #odds ratio
```

```
## (Intercept)         ACD
##   2.7713073   0.7126031
```

```r
exp(confint(ACD)) # 95% confidence interval
```

```
##                  2.5 %     97.5 %
## (Intercept) 0.06646657 125.804609
## ACD         0.13874891   3.552942
```

```r
LT <- glm(Barrett_APE~ LT,family=binomial(link='logit'),data=data)
p <- summary(LT)
p$coefficients # p-value for coefficients
```

```
##               Estimate Std. Error    z value  Pr(>|z|)
## (Intercept)  1.2909624  3.0080536  0.4291687 0.6678005
## LT          -0.2122378  0.6034011 -0.3517359 0.7250364
```

```r
exp(coef(LT)) #odds ratio
```

```
## (Intercept)          LT
##   3.6362845   0.8087723
```

```r
exp(confint(LT)) # 95% confidence interval
```

```
##                   2.5 %      97.5 %
## (Intercept) 0.009849941 1422.602633
## LT          0.244545384    2.651876
```

```r
W2W <- glm(Barrett_APE~ W2W,family=binomial(link='logit'),data=data)
p <- summary(W2W)
p$coefficients # p-value for coefficients
```

```
##               Estimate Std. Error   z value   Pr(>|z|)
## (Intercept)  8.7646727  4.8122867  1.821311 0.06855953
## W2W         -0.7511586  0.4230664 -1.775510 0.07581376
```

```r
exp(coef(W2W)) #odds ratio
```

```
##  (Intercept)          W2W
## 6403.9653940    0.4718196
```

```r
exp(confint(W2W)) # 95% confidence interval
```

```
##                 2.5 %       97.5 %
## (Intercept) 0.6585595 1.208902e+08
## W2W         0.1987083 1.058530e+00
```

```r
CCT <- glm(Barrett_APE~ CCT,family=binomial(link='logit'),data=data)
p <- summary(CCT)
p$coefficients # p-value for coefficients
```

```
##                  Estimate  Std. Error    z value  Pr(>|z|)
## (Intercept)  1.413291447 2.827187103  0.4998931 0.6171503
## CCT         -0.002130788 0.005100713 -0.4177431 0.6761349
```

```r
exp(coef(CCT)) #odds ratio
```

```
## (Intercept)         CCT
##   4.1094592   0.9978715
```

```r
exp(confint(CCT)) # 95% confidence interval
```

```
##                   2.5 %      97.5 %
## (Intercept) 0.01600552 1127.500987
## CCT         0.98782632    1.007925
```

```r
sex <- glm(Barrett_APE~ sex,family=binomial(link='logit'),data=data)
p <- summary(sex)
p$coefficients # p-value for coefficients
```

```
##               Estimate Std. Error  z value  Pr(>|z|)
## (Intercept) 0.1026542  0.2267538 0.452712 0.6507561
## sexMale     0.4569616  0.4270476 1.070049 0.2845975
```

```r
exp(coef(sex)) #odds ratio
```

```
## (Intercept)     sexMale
##    1.108108    1.579268
```

```r
exp(confint(sex)) # 95% confidence interval
```

```
##                 2.5 %   97.5 %
## (Intercept) 0.7104070 1.734335
## sexMale     0.6914235 3.726717
```

```r
age <- glm(Barrett_APE~ age,family=binomial(link='logit'),data=data)
p <- summary(age)
p$coefficients # p-value for coefficients
```

```
##                Estimate Std. Error    z value  Pr(>|z|)
## (Intercept)  1.10623680 1.54873061  0.7142861 0.4750503
## age         -0.01355354 0.02389826 -0.5671349 0.5706226
```

```r
exp(coef(age)) #odds ratio
```

```
## (Intercept)         age
##   3.0229609   0.9865379
```

```r
exp(confint(age)) # 95% confidence interval
```

```
##                 2.5 %    97.5 %
## (Intercept) 0.1468334 66.555542
## age         0.9406867  1.033801
```

5.42 Multivariate logistic regression

```r
formula=Barrett_APE~ AL+K_mean+ACD+LT+W2W+CCT+sex+age
fit1 <- glm(formula,family=binomial(link='logit'),data=data)
p <- summary(fit1)
p$coefficients # p-value for coefficients
```

```
##                 Estimate   Std. Error    z value  Pr(>|z|)
## (Intercept) 21.398493237 15.087343846  1.4183075 0.1561010
## AL          -0.690113085  0.369375591 -1.8683235 0.0617170
## K_mean       0.043816086  0.178419364  0.2455792 0.8060080
## ACD         -0.284424359  0.998599487 -0.2848233 0.7757796
## LT          -0.157893060  0.700827159 -0.2252953 0.8217496
## W2W         -0.349843684  0.506834188 -0.6902527 0.4900353
## CCT         -0.003804481  0.005621674 -0.6767524 0.4985631
## sexMale      0.998859629  0.492573005  2.0278408 0.0425765
## age         -0.006093766  0.026995081 -0.2257362 0.8214066
```

```r
exp(coef(fit1)) #odds ratio
```

```
##  (Intercept)           AL       K_mean          ACD           LT          W2W
## 1.964480e+09 5.015194e-01 1.044790e+00 7.524473e-01 8.539411e-01 7.047983e-01
##          CCT      sexMale          age
## 9.962027e-01 2.715184e+00 9.939248e-01
```

```r
exp(confint(fit1)) # 95% confidence interval
```

```
##                    2.5 %       97.5 %
## (Intercept) 0.0004477557 3.838707e+22
## AL          0.2338314947 1.009777e+00
## K_mean      0.7321838730 1.484620e+00
## ACD         0.1019688849 5.349845e+00
## LT          0.2138162758 3.421938e+00
## W2W         0.2544074113 1.890354e+00
## CCT         0.9851453391 1.007275e+00
## sexMale     1.0623625497 7.426548e+00
## age         0.9424186593 1.048635e+00
```

5.43 nomogram construction

```
dd <- datadist(data)
options(datadist="dd")
formula=Barrett_APE~ age+sex+CCT+W2W+LT+ACD+K_mean+AL
fit <- lrm(formula = formula,data=data,x=T,y=T)
nom <- nomogram(fit,
         fun=function(x)1/(1+exp(-x)),
         fun.at = c(0.001,0.01,0.05,seq(0.1,0.9,by=0.1),0.95,0.99,0.999),
         lp=F,
         funlabel = "Pr(Barrett II)")
plot(nom)
```

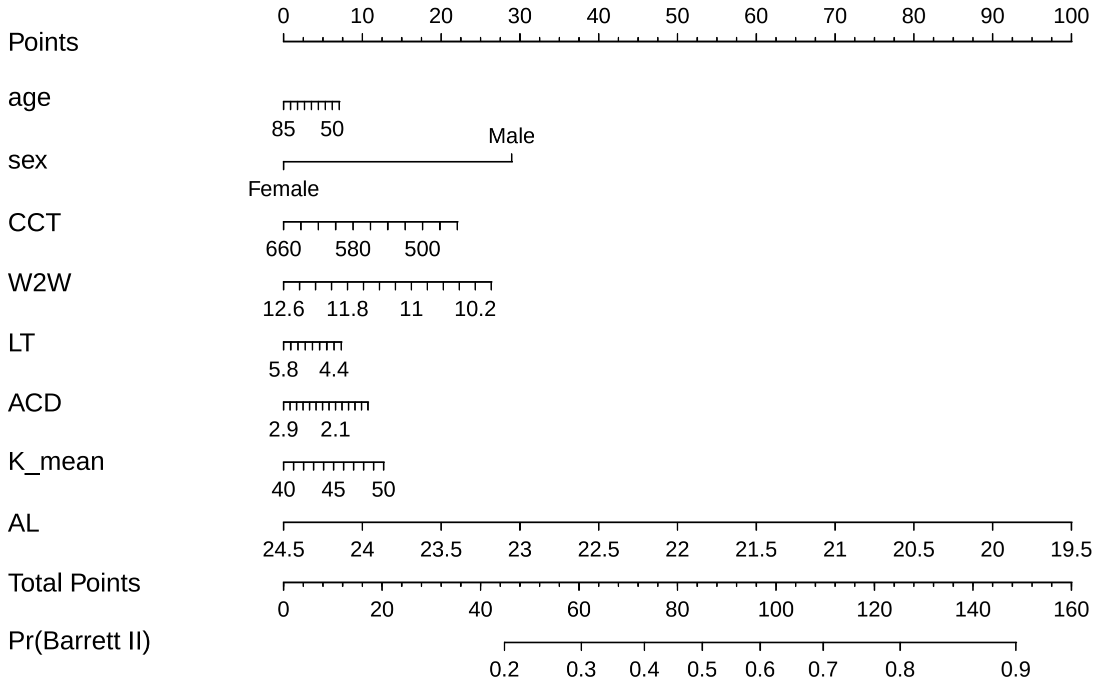

5.44 Accuracy of the model

We plot a ROC curve here, and the area under the ROC curve was used to represent the accuracy of nomogram model.

```
fit1 <- glm(formula = formula,family=binomial(link='logit'),data=data)
pre = predict(fit1,type="response")
modelroc <- pROC::roc(data$Barrett_APE,pre,
                      ci=T, # arguments for ci
                      boot.n=300,
```

```
                            ci.alpha=0.95,
                            stratified=T)
plot(modelroc,
     print.auc=T,
     auc.polygon=T,
     grid=c(0.1,0.2),
     grid.col=c("#FFF0F5","#FFC0CB"),
     max.auc.polygon=T,
     auc.polygon.col="#CCCCFF",
     print.thres=F)
```

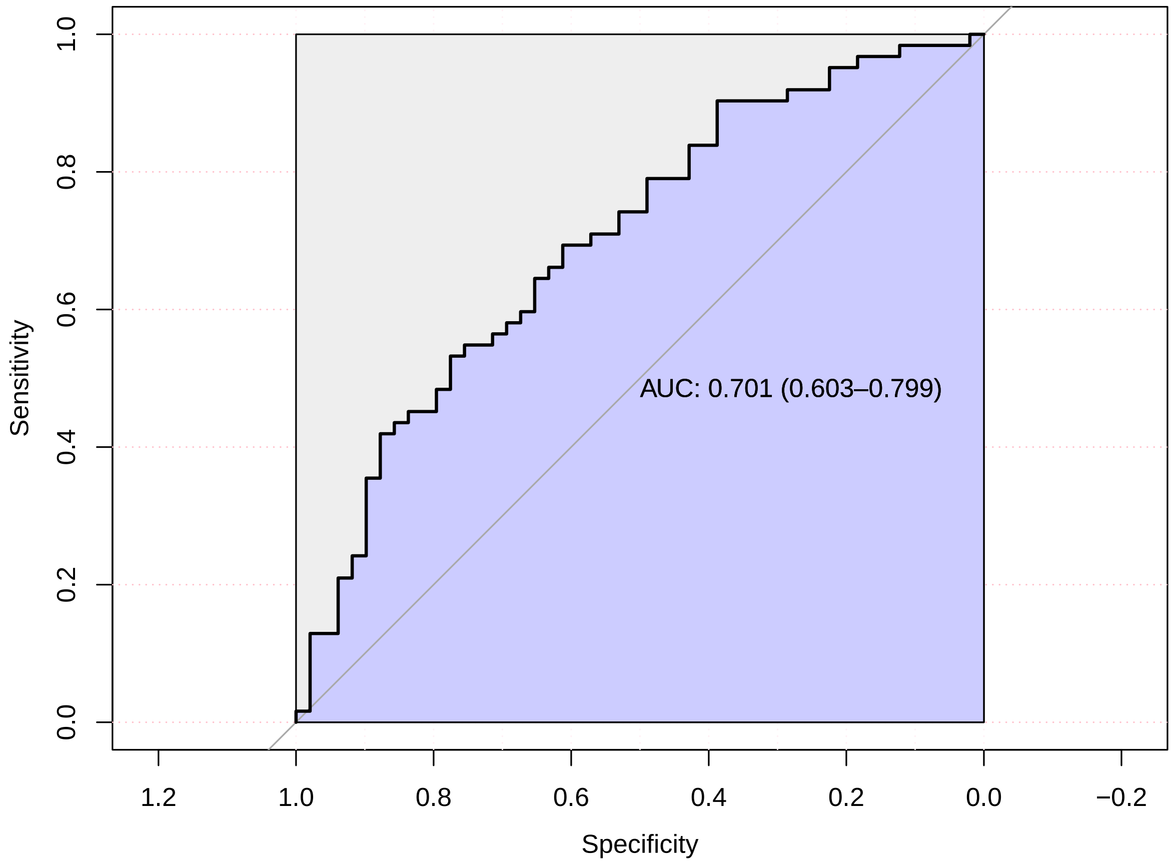

5.45 Calibration plot and Hosmer-Lemeshow test

Nomogram model was validated using calibration curve with bootstrap method, and Hosmer-Lemeshow goodness-of-fit test was conducted.

```
nom <- lrm(formula = formula,data=data,x=T,y=T)
cal <- calibrate(nom,method="boot",B=100)
par(mar=c(8,4,1,2))
plot(cal)
```

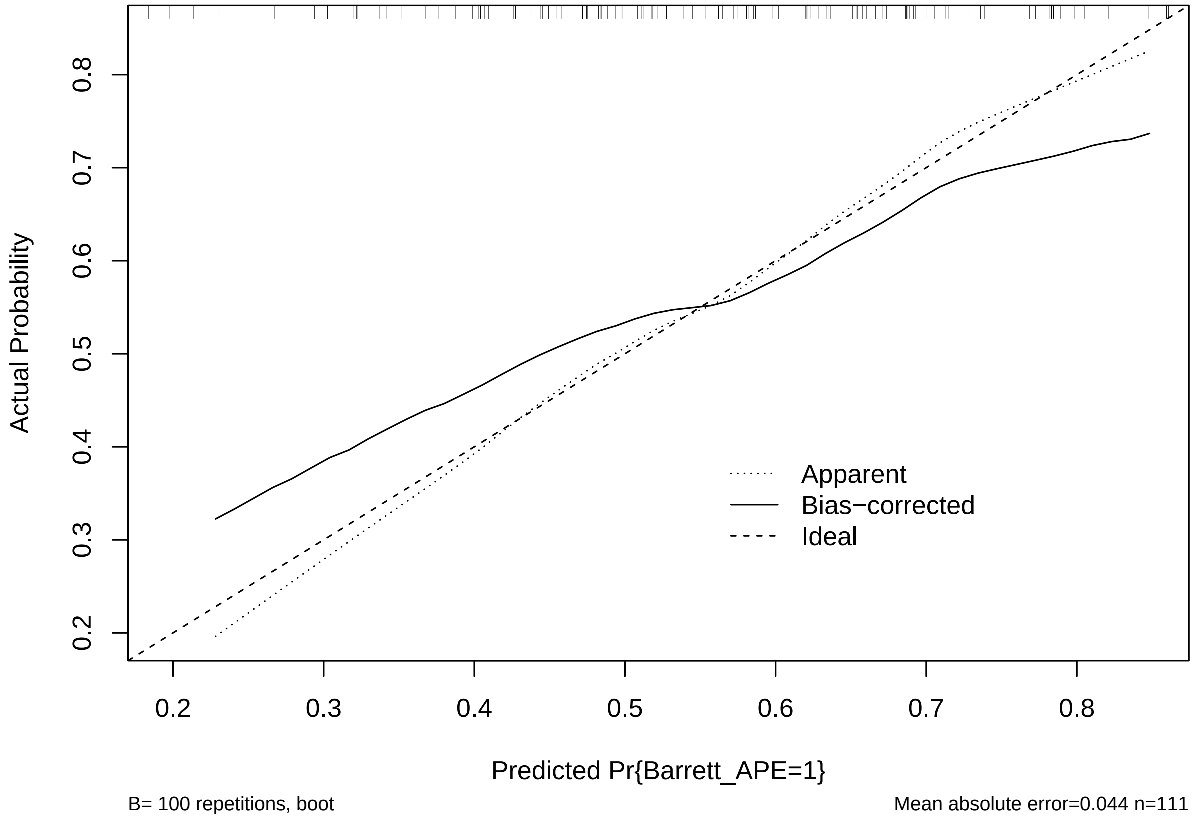


```
##
## n=111   Mean absolute error=0.044   Mean squared error=0.00253
## 0.9 Quantile of absolute error=0.079
```

```
# HL-test
h.test <- hoslem.test(fit1$y, fitted(fit1), g=10)
h.test
```

```
##
##  Hosmer and Lemeshow goodness of fit (GOF) test
##
## data:  fit1$y, fitted(fit1)
## X-squared = 5.3076, df = 8, p-value = 0.7243
```
